# The landscape of artificial intelligence tools and platforms for evidence synthesis: a scoping review

**DOI:** 10.1186/s13643-025-02842-y

**Published:** 2026-02-10

**Authors:** M. Sharmila A. Sousa, Sasha Peiris, Mabel F. Figueiró, Michelle M. Haby, Ana Cyntia Baraldi, Ludovic Reveiz, João Paulo Souza

**Affiliations:** 1 Department of Medicine, Department of Morphology and Genetics, Escola Paulista de Medicina, Universidade Federal de São Paulo, São Paulo, Brazil; 2 Science and Knowledge for Impact Unit, Evidence and Intelligence for Action in Health, Pan American Health Organization, Washington, DC, USA; 3 Hcor – Associação Beneficente Síria, São Paulo, Brazil; 4 Faculty of Biological and Health Sciences, University of Sonora, Hermosillo, Mexico; 5 Evidence and Intelligence for Action in Health, BIREME, Pan American Health Organization, Brasilia, Brazil

## Abstract

Evidence synthesis (ES) involves rigorous, reproducible methodologies, which are increasingly being presented as ‘Living’ systematic reviews. As such, ES are critical to evidence-informed decision-making processes, such as the development, implementation, evaluation and monitoring of health technology assessments, practice guidelines and policies. However, the ES process is time-intensive, typically requiring months or years and extensive manual effort. Technological advancements, particularly artificial intelligence (AI), offer opportunities to automate various ES steps, potentially increasing efficiency and reducing costs. AI tools and platforms, including large language models (LLMs), facilitate faster ES through advanced natural language processing (NLP) capabilities. Despite their potential, AI tools have limitations, including risks of automation bias and lack of true semantic understanding, requiring careful evaluation to ensure trustworthiness. We conducted the first scoping review to update and map all data science tools, including LLMs, which are either being developed and/or deployed to optimise ES steps and assess their impact in both low- and middle-income countries (LMICs) and high-income countries (HICs). Our scoping review identified 137 studies and 388 of such AI tools and platforms to respond to the World Health Organization’s call for safe and ethical AI in health, documenting the current landscape to identify barriers and facilitators to equitable and sustainable access for glocal researchers. We further outline three recommendations: (1) promote collaborative AI platforms ensuring equity of access to include gap regions identified (Latin America, Africa, Middle East), (2) establish evaluation standards for methods testing and reporting, and (3) emphasise human input and multidisciplinary capacity building for developing and implementing AI tools in ES.

**Supplementary Information:**

The online version contains supplementary material available at 10.1186/s13643-025-02842-y.

## Background

There is an abundance of literature and information that is produced daily in the form of qualitative and quantitative studies, reports and documents. In an era of evidence-based practice and policy, it is imperative that decision-makers have the best available evidence synthesised from multiple sources [1]. There are many interpretations on what ‘evidence synthesis’ (ES) encompasses. For this paper, we consider ES as identifying and combining research that has already been collected through primary [2] and secondary research. This synthesised evidence, based on rigorous, reproducible and transparent methodologies, is often but not exclusively published as systematic literature reviews (SLRs), and more recently as living systematic reviews (or Living ES) [3], and can also include rapid reviews [4], scoping reviews [5, 6] and qualitative ES [7], among other research ES methods.

ES provides the evidence base for the development of practice guidelines, health technology assessment, list of essential medicines and other technologies, coverage decisions, policy documents, reports and the material for debate and discussions in health care on specific issues for various purposes [8] and systems management decision and policy-making processes [9]. In reality, ES is time-intensive typically taking months or years to conduct a SR [10] and requiring a multi-person research team [11] (e.g. it takes more than 200 person-hours of manual work to sift through titles and abstracts of potential studies per systematic review, and only a minority of reviews are updated within a couple of years of publication) [12, 13]. Technological advances have enabled the full or semi-autonomous performances of one or more of the ES steps — i.e. planning (issue identification and questions determination), protocol development and registration, search for studies in online databases, screening studies, studies selection according to inclusion/exclusion criteria, data extraction, critical quality appraisal, data synthesis (e.g. meta-aggregation, meta-analysis), summary of findings, discussion and conclusions, reference management, figures and tables, ES publication, dissemination and registration in specific databases — with the aim to reduce the workload, making the process more efficient, cost-effective and sustainable [10].

### Automation of ES steps

Artificial intelligence (AI) tools and platforms, including specialised systems and large language models (LLMs), can be deployed to facilitate human efforts to synthesise evidence faster for evidence-informed guidance and policies. AI tools and platforms, such as the *Epistemonikos database*, allow efficient and intuitive searching for systematic reviews and individual studies [14]. Algorithms are used for entity recognition, information extraction, pattern detection, among other automation strategies of ES steps [15, 16]. The application of AI for ES should be rigorous and use codified methods that reduce risk of bias and ensure trustworthiness. Nevertheless, AI tools have under-delivered [17]. LLMs, as one type of generative foundation AI models, are trained through a variety of deep learning (DL) and neural networks (NNs) algorithms among other data science methods to recognise, translate and/or summarise massive amounts of written human language and textual data sets, including websites, articles and books, to generate text, translate between languages and write many types of content, with the goal of generating new text that closely resembles human responses [18]. As such, LLMs constitute some of the most advanced and accessible natural language processing (NLP) solutions today that imitate understanding, processing and production of human communication [19, 20].

Nevertheless, LLMs do not understand the semantic meaning of a sentence in a linguistic sense (i.e. cause and effect and the relationships between objects) but rather calculate mathematically what the most likely next word should be based on the input to the model. This constitutes a critical limitation that users should carefully examine to avoid risk of automation bias (i.e. misplacing trust in their outputs) and anthropomorphism (i.e. establishing human-like rapport with LLMs, further exacerbating automation bias) [20]. Such awareness is key when using LLMs either to improve access to health information and its synthesis, as a decision-support tool, or to enhance diagnostic capacity in under-resourced settings to protect people’s health and reduce inequity [21]. There is much discussion that the advent of powerful LLMs will accelerate the (semi-)automation of SRs and accelerate the scaling and adoption of Living ES [22]. The use of AI in high-income countries (HICs) is viewed as a promising tool for improving health systems [23] but it is still considered a luxury in low-resourced settings due to the many operational, managerial and process challenges [24]. Therefore, access costs remain a key issue, globally. Proprietary AI tools and platforms are owned by companies and can only be used by customers that purchase a license, which may further restrict how they can be deployed. Whereas, open source ones comprise free code and underlying architecture that developers and researchers can access, use for any purpose, modify, improve and distribute them [18]. Please refer to Table 1 for operational definitions of data science terminology used throughout the manuscript. NB: We listed these definitions in an increasing order of complexity.

**Table 1:** Data science terminology

Data mining (DM) is a multidisciplinary field at the intersection of database technology, statistics, machine learning and pattern recognition that profits from all these disciplines [18]
Text mining (TM) can be defined as ‘the process of discovering knowledge and structure from unstructured data (i.e. text)’ [25]
Machine learning (ML) consists of computer algorithms that learn from sample inputs and apply that learning to make predictions on data or classify data into categories. Therefore, these algorithms learn to perform a specific task through statistical modelling of typically large amounts of data without being explicitly programmed. Such learning can be supervised by humans or unsupervised [8]
Deep learning (DL) is a subset of ML, comprising computational models and algorithms that imitate the biological neural networks’ architecture in the brain, therefore, being considered ‘artificial neural networks’ (ANNs). As such, whenever such ANNs receive new information, they try to compare it with already known information as an attempt to understand it, further deciphering it through labelling and assigning items to various categories. ‘Deep’ refers to the number of layers in any ANN. Furthermore, there are three types of layers: input layer (receives input data), output layer (produces result from data processing) and hidden layer (extracts patterns within the data). A deep ANN differs from a superficial ANN (single hidden layer) by having a large number of hidden layers — this means a DL is able to perform more complex tasks. DL also works exceptionally well on unstructured data and has higher accuracy than ML but requires a huge volume of training data, along with expensive hardware and software [26]
Neural networks (NNs) are a type of ML process called DL, which is a computing system that uses interconnected nodes or (artificial) neurons in a layered structure that resembles the human brain to process data and produce an output. It creates an adaptive system that computers use to learn from their mistakes and improve continuously. Thus, ANNs attempt to solve complicated problems, like summarising documents or recognizing faces, with greater accuracy, helping computers make complex decisions with limited human assistance [27]
Natural language processing (NLP) is the study of computer programs that take natural, or human, language as input [28]
Large language models (LLMs) include some of the most rapidly expanding AI platforms such as ChatGPT and DALL-E (OpenAI, San Francisco, CA, USA), BARD and LaMDA (Google, Mountain View, CA, USA), Llama/Llama- 2 (Meta, Menlo Park, CA, USA) and many others that imitate understanding, processing and producing of human communication [15]. As one type of generative foundation AI models, LLMs are trained through a variety of DL and NNs algorithms among other data science methods to recognise, translate and/or summarise massive amounts of written human language and textual data sets, including websites, articles and books, to generate text, translate between languages and write many types of content, with the goal of generating new text that closely resembles human responses [18]. As such, LLMs constitute some of the most advanced and accessible NLP solutions today that imitate understanding, processing and production of human communication [19, 20]

### Evidence gap

We conducted a preliminary search for existing scoping reviews on the topic of AI tools and platforms in PubMed, Embase, and Epistemonikos in October 2023 and found two scoping reviews and a mapping review. Two studies systematically mapped the literature on the availability of automation tools that assist the SR process [29, 30], and one study examined the use of AI in automation of biomedical literature analyses [31]. These studies were conducted in 2021 and 2022, but the last couple of years have seen a substantial increase in investments dedicated to the development of AI tools [32]. There is no comprehensive documentation evaluating (e.g. for impact on risk of bias, time-savings) AI tools and platforms (which included LLMs) deployed for ES. There are also questions related to equity of access and affordability for researchers globally. As such, our objectives were twofold. First, we aimed to update and map all levels of automation, including AI tools and platforms, especially LLMs, which are being deployed and/or developed to optimise ES. Second, we wanted to identify potential analyses of their impact on any of the ES steps at low- and middle-income countries (LMICs) and HICs, given the World Health Organization calls for safe and ethical AI in health [33]. For such purposes, we developed a scoping review. Here, we describe our findings on this update mapping exercise to include LLMs.

## Methods

This scoping review followed the framework outlined by Arksey and O’Malley [5], and the adopted updated recommendation by Levac and colleagues [6], with the exception of the final ‘stakeholders’ consulting’ step, which was not considered for this review due to time constraints. Nevertheless, JPS and MSAS have some experience in testing development and implementation of AI tools and platforms for ES automation, respectively. This review was conducted to comply with the Preferred Reporting Items for Systematic reviews and Meta-Analyses (PRISMA) extension for Scoping Reviews (PRISMA-ScR) reporting guidelines [34], summarised in Supplementary Table S1. PROSPERO does not currently accept registrations for scoping reviews, literature reviews or mapping reviews. Therefore, we registered the scoping review protocol *https://osf.io/ayb2r/?view_only=b682b48a91d0497e8451bf6cce36edd5* at the Open Science Framework (OSF) platform^1^. The review began in October 2023 and ended by April 2024.

Our scoping review questions was as follows:

- Which levels of automation, including AI tools and platforms, especially LLMs, are available and where are they being used to optimise and accelerate any of the ES steps in the health field?
- Which levels of automation, including AI tools and platforms, especially LLMs, are currently being developed to optimise and accelerate which ES steps?
- Which levels of automation, including AI tools and platforms, especially LLMs, are being applied to which ES steps?
- What are the barriers and facilitators to each of these levels of automation, including AI tools and platforms, especially LLMs, in optimising and accelerating (i.e. semi-automatising with human validation) any of the ES steps at LMICs, as compared to HICs?

### Information sources

We conducted a comprehensive search of PubMed, Embase, Cochrane Library, the Pan American Health Organization (PAHO), Virtual Health Library (VHL), Web of Science and Google Scholar (to include grey literature such as full-text conference proceedings articles, abstracts and university thesis and dissertations databases). The search strategy was piloted and checked for relevance of keywords and databases by two reviewers (MS AS, SP). In all retrieved publications, an analysis of the words contained within the title and abstracts, as well as index terms, was done to develop a full search strategy with the help of an experienced medical librarian (MFF.), followed by a second search using all the identified keywords and index terms, as detailed in the scoping review *protocol*. Reference lists of all selected studies were screened to look for additional sources. There was no language restriction. Studies not in English, Spanish or Portuguese were translated by reviewers with the aid of Google Translate. All references identified were imported into the reference manager software Mendeley^©^ for management and removal of duplicates between 26 November 2023 (all databases except Google) and 3 December 2023 (only Google). We re-ran the PubMed search only on 22 July 2024 just before the final analyses. From the extra records retrieved (approximately 100), none was eligible for inclusion as we were unable to access them online.

### Screening, study selection and eligibility criteria

The study selection followed a two-step screening process, consisting of a title and abstract screening, followed by review of the full text of potentially eligible studies. The title and abstract screening was performed in parallel and independently by four researchers working in pairs (MFF, MSAS, MH and SP) using Rayyan [35]. Discrepancies were resolved through consensus. Full-text screening and data extraction were performed in parallel and independently by these four researchers, after sample calibration and consensus. We considered all publications that synthesised primary and/or secondary research studies, which included experimental, observational, qualitative and mixed-methods study designs, as well as systematic, rapid reviews, other types of nonsystematic literature review and full-text grey literature. Publications should describe, discuss, comment or critically analyse how all levels of automation, including AI tools and platforms, especially LLMs, are being developed and/or deployed to optimise and/or accelerate (and semi-automatise with human validation) any of the ES steps for all types of primary and secondary ES designs, as previously described, per country. This also included ES products being used to inform front-line decision-making for health care and systems purposes at local level.

We did not consider publications of case studies of all levels of automation, including AI tools and platforms, especially LLMs, being deployed to optimise and/or accelerate (and semi-automatise with human validation) the generation of primary research findings for any specific disease and/or health issue. As such, we did not include studies of automation of randomised-controlled trials data, if it was not a methodological study to verify data science methods for such automation of ES steps. Although we did not exclude studies that did not outline data science methods being deployed for each AI tool and platforms being described, analysed and/or discussed, specifically, we managed to classify each study design under greater data science umbrella methods (i.e. DM, TM, ML, DL, NNs, NLP and LLMs). Finally, we excluded all studies that did not specify data science tools and the ES step(s) it was either being developed and/or implemented to optimise.

### Data extraction, analysis and synthesis

A data extraction form (Microsoft Excel) was developed using key themes from the research questions. Relevant data was extracted from all included studies in the scoping review by four independent reviewers. The data extraction tool was piloted and revised as necessary during the process of extracting data from each study. Disagreements between reviewers were resolved through discussion, and a review author (J. P. S.) resolved one extraction discrepancy, improving the data extraction form. We also did not contact study authors for any missing or incomplete data. Data extracted comprised the following: author, year, study title, country(ies), region(s), study design and objective(s); results (data science method(s), data science tool(s), ES step(s), other data science tool(s), development and/or implementation finding(s) and recommendations, tool access cost (i.e. proprietary, open source/free or mixed) and automation development stage ((i.e. developing or implementing)); and study limitations, conclusions and proprietary developers (i.e. potential conflicts of interest). Additional information extracted included development and implementation barriers and facilitators at LMICs and HICs (as we considered countries from authors’ institutional affiliation(s) when studies did not describe location of AI tools’ and platforms’ development and/or implementation). The extracted data was qualitatively analysed and meta-aggregated [7] by MSAS in Supplementary Table S2.

#### Findings

##### Study selection and characteristics

**Fig. 1:**
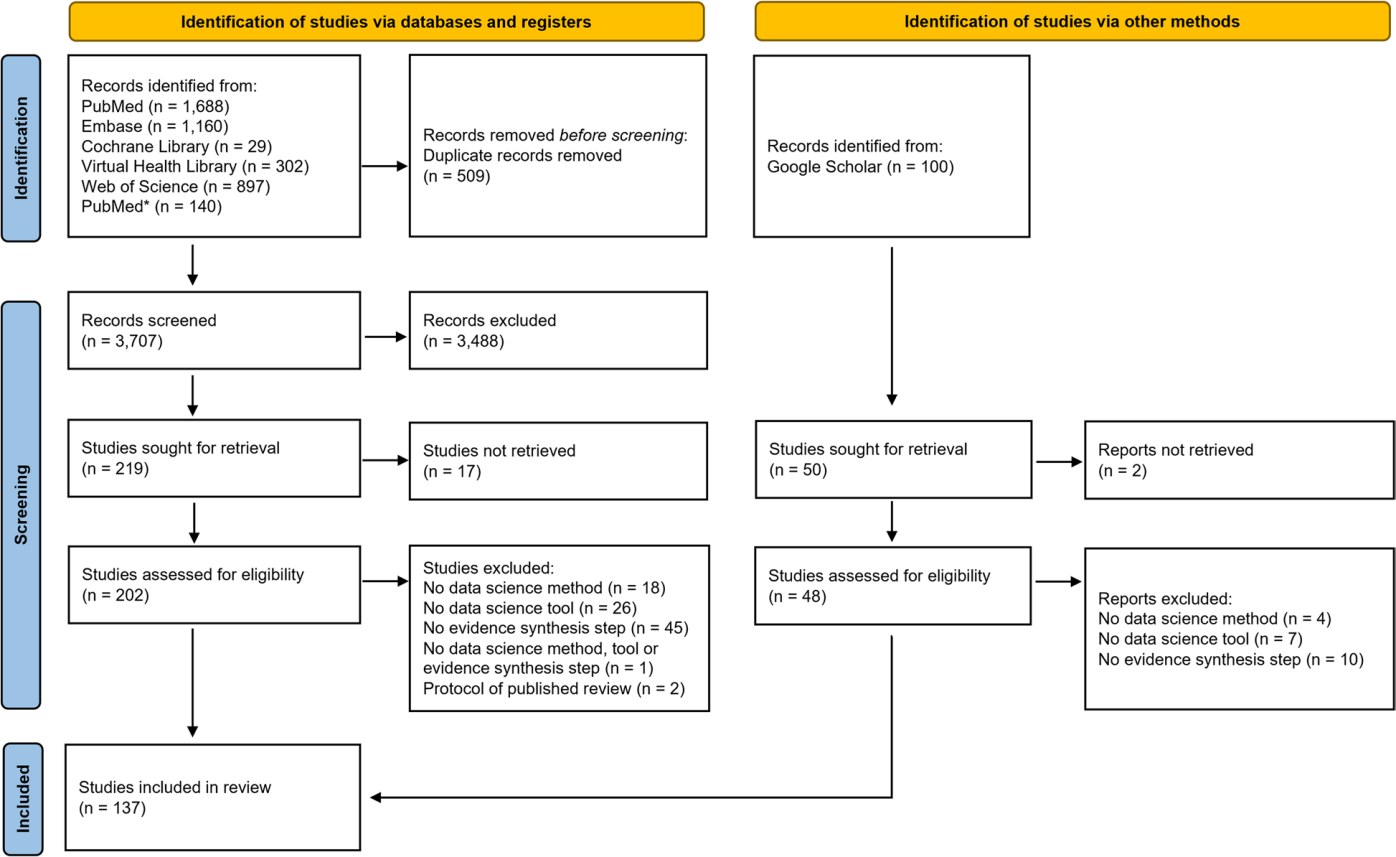
PRISMA systematic review flow chart of records identification, study screening and selection *July/2024 (PubMed search update only)

The search strategies for all aforementioned electronic bibliographic databases retrieved 4176 results. After excluding 509 duplicates, 3667 studies were formally screened for title and abstract against eligibility criteria. After full-text screening of 248 studies against the eligibility criteria, we included 133 of these studies. We report the number of records identified from each database or register searched (rather than the total number across all databases/registers). We did not use automation tools for screening; therefore, all records were manually excluded by a human. Search update was performed in July/2024 (PubMed only), identifying 140 studies, which were screened for title and abstract against eligibility criteria. From these, we included extra 4 studies, giving 137 studies in total. The selection flowchart outlining the reasons for exclusion is presented in Fig. 1.

**Fig. 2:**
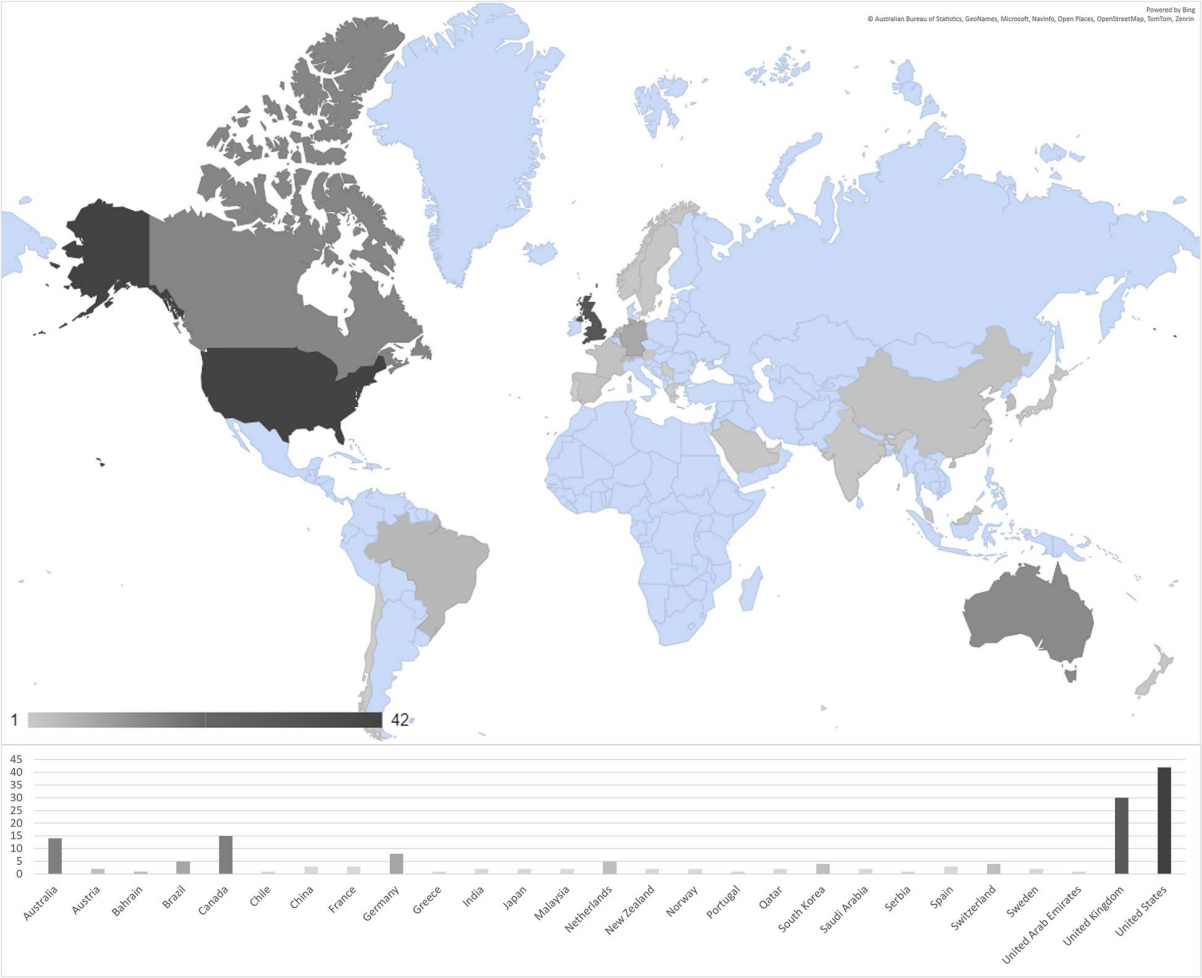
Map of number of publications per country (identified)

**Fig. 3:**
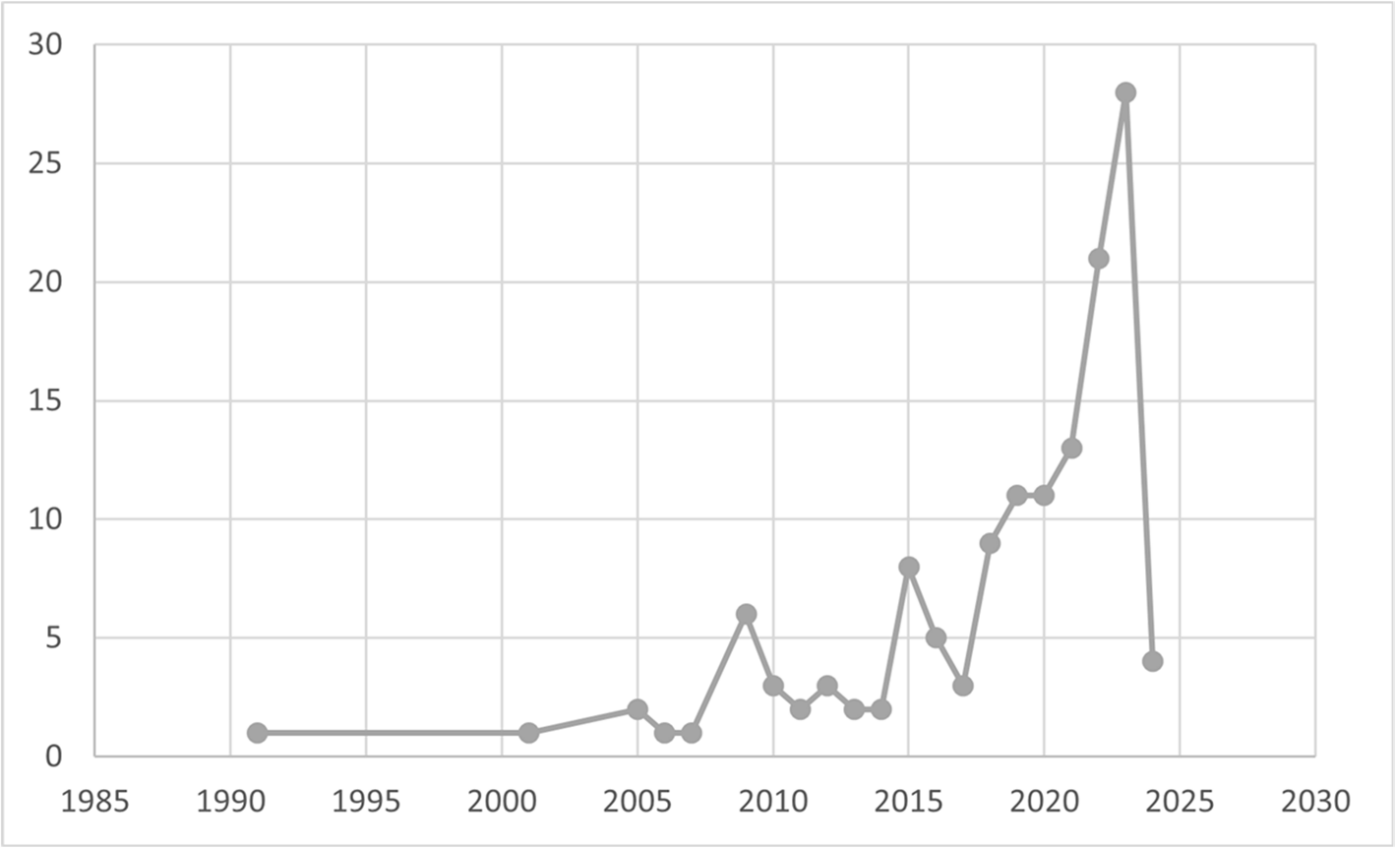
Number of publications per year

We identified evidence gap areas where there is paucity of data, geographically and chronologically, as summarised in Figs. 2 and 3, respectively. We further outline that search did not cover the entirety of 2024 (only until July) and was implemented only for PubMed, hence the (potential) drop in results for the final year in Fig. 3.

**Fig. 4:**
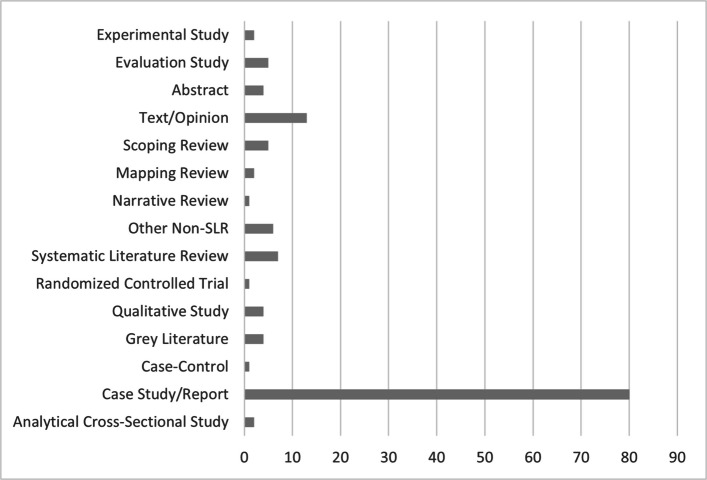
Number of publications per study design

We have also identified the types of primary and secondary study designs, as summarised in Fig. 4.

We categorised all AI tools and platforms we identified, according to country(ies) where they are being either developed and/or implemented to automatise/optimise and/or accelerate any of the ES step(s) for each of the types of primary and secondary study designs (summarised in Supplementary Table S3). As such, we were able to observe a series of patterns and trends that answered our review questions, as follows.

### Global landscape of artificial intelligence tools and platforms for ES automation

Our review updates the global landscape of data science methods and approaches that are being deployed to develop and/or implement various types of AI tools and platforms to also include LLMs, as summarised in Table 2. We identified that global authors have reported either developing and/or implementing less DM approaches (*n* = 8/137) to automatise only a few ES steps than TM models (*n* = 28/137) and to automatise all ES steps. Our review has also identified that ML (*n* = 65/137) is the most deployed data science method for both development and/or implementation of all ES steps optimisation, historically. DL (*n* = 6/137) remains the data science method least deployed, whereas NNs (*n* = 9/137) are being increasingly used to automatise still fewer ES steps. Meanwhile, NLP (*n* = 20/137) has been used more broadly to optimise all ES steps, as well as LLMs (*n* = 25/137). Our review adds to this landscape, by outlining those AI tools and platforms that only recently started being deployed and further revolutionising, in ways, the ES field, given the speed and broad scope of their development and implementation.

**Table 2:** Global data science methods being developed and/or implemented for ES steps automation

	**Data science method**						
ES step	**DM** (*n* = 8/133)	**TM** (*n* = 27/133)	**ML** (*n* = 63/133)	**DL** (*n* = 5/133)	**NNs** (*n* = 8/133)	**NLP** (*n* = 20/133)	**LLMs** (*n* = 22/133)
Study protocol	*n* = 2 [36, 37]	*n* = 5 [38–42]	*n* = 3 [40, 41, 43]	None	*n* = 1 [44]	*n* = 1 [45]	*n* = 4 [46–49]
Search	*n* = 2 [41, 50]	*n* = 16 [31, 38–41, 51–61]	*n* = 20 [8, 17, 30, 40, 41, 55, 58, 62–74]	*n* = 3 [62, 75, 76]	*n* = 2 [8, 58]	*n* = 12 [8, 17, 30, 31, 45, 55, 62, 66, 69, 76–78]	*n* = 4 [46, 48, 49, 79]
Reference management	None	None	*n* = 6 [29, 31, 40, 41, 68, 80]	None	None	*n* = 1 [45]	*n* = 3 [46, 48, 49]
Deduplication	None	*n* = 2 [40, 60]	*n* = 9 [29–31, 40, 41, 68, 81–83]	None	None	*n* = 2 [30, 45]	*n* = 3 [46, 48, 49]
Screening	*n* = 1 [84]	*n* = 14 [31, 38, 40, 41, 53, 55, 57, 58, 60, 85–89]	*n* = 47 [4, 13, 21–23, 29, 35, 36, 38, 40, 50, 53, 56–58, 61–67, 79, 81–102]	*n* = 4 [61, 62, 76, 90]	*n* = 4 [8, 58, 61, 91]	*n* = 14 [8, 17, 30, 31, 45, 55, 62, 69, 76, 89, 92–95]	*n* = 9 [46, 48, 49, 61, 79, 96–99]
Selection	None	*n* = 6 [36, 38, 40, 57, 61, 100]	*n* = 16 [29, 36, 40, 41, 64, 67–70, 72–74, 101–104]	*n* = 1 [76]	None	*n* = 4 [36, 45, 69, 76]	*n* = 2 [46, 48]
Extraction	*n* = 3 [105–107]	*n* = 12 [31, 38, 40, 55, 57, 58, 61, 106, 108–111]	*n* = 18 [8, 17, 29–31, 36, 40, 41, 44, 55, 58, 61, 64, 68, 71, 112–114]	*n* = 3 [44, 61, 75]	*n* = 5 [8, 41, 44, 58, 115]	*n* = 7 [8, 17, 30, 31, 44, 45, 112]	*n* = 8 [44, 46, 48, 49, 79, 116–118]
Critical quality appraisal	None	*n* = 3 [38, 40, 57]	*n* = 13 [8, 29, 40, 41, 43, 63, 64, 66, 70, 119–122]	None	*n* = 3 [8, 41, 122]	*n* = 6 [8, 36, 45, 55, 66, 121]	*n* = 4 [46, 48, 49, 123]
Meta-analysis	None	None	*n* = 7 [17, 29, 31, 40, 41, 64, 66]	None	None	*n* = 3 [31, 66, 89]	*n* = 3 [46, 48, 49]
Figures visualisation	None	None	*n* = 5 [29, 40, 41, 66, 68]	None	None	*n* = 2 [45, 66]	*n* = 3 [46, 48, 49]
Summary/synthesis	*n* = 2 [106, 107]	*n* = 7 [38, 40, 53, 55, 57, 58, 106]	*n* = 13 [17, 29, 31, 36, 40, 41, 43, 55, 58, 63, 64, 68, 71]	*n* = 1 [75]	*n* = 2 [36, 58]	*n* = 4 [17, 36, 45, 55]	*n* = 12 [36, 46–49, 79, 118, 124–128]
Publication	None	*n* = 1 [40]	*n* = 4 [40, 41, 64, 71]	None	None	*n* = 1 [45]	*n* = 8 [46–49, 117, 129–131]
SLR database	None	*n* = 1 [40]	*n* = 3 [31, 40, 41]	None	None	*n* = 1 [45]	*n* = 3 [46, 48, 49]

*ES* Evidence synthesis, *DM* Data mining, *TM* Text mining, *ML* Machine learning, *DL* Deep learning, *NNs* Neural networks, *NLP* Natural language processing, *LLMs* Large language models

We have also synthesised all AI tools and platforms under their commercial names, outlining which types of data science methods they deploy to develop and/or implement ES steps automation, as summarised in Supplementary Table S4. We have updated Jimenez and colleagues infographic [30] on ML and NLP tools and techniques for such purposes and added the new findings from our review on all other AI tools and platforms deploying DM, TM, DL, NNs and LLM (single or mixed) approaches or both developing and implementing ES steps automation. We also outline which data science method is being deployed by each tool and platform, those that are either open source/free for access (free) or proprietary ($), requiring payment for access, as well as whether they are being only developed or also implemented (*). We provide a hyperlink to access further information regarding each tool and platform — all according to information found in the 137 included studies and reference lists (NB: those without a link have been identified in one of the ES included whose reference is listed in Supplementary Table S3). Finally, we have also outlined regional specialisation, as summarised in Supplementary Findings.

### HICs leaders in development and implementation

Both the USA (*n* = 28/42) and UK (*n* = 23/31), followed by Canadian- (*n* = 12/15) and Australian-based studies (n = 12/14), are the HICs reporting more development and/or implementation of AI tools and platforms, deploying chiefly *TM* (*n* = 17/82) [40–42, 52–54, 56–60, 86–89, 108, 110], *ML* (*n* = 39/82) [8, 17, 29, 40, 58, 62, 65, 66, 71–73, 80–83, 88, 89, 92, 101–103, 113, 114, 119, 121, 132–145], *NLP* (*n* = 14/82) [8, 17, 44, 62, 66, 76, 77, 88, 89, 93–95, 121, 146] and *LLMs* (*n* = 10/82) [79, 97, 98, 116, 124–129] of the data science method types to automatise predominantly the following ES steps: *study protocol* (*n* = 8/82) [37, 40–42, 147–150], *search* (*n* = 33/82) [8, 17, 29, 40, 41, 45, 50, 52–54, 56–60, 62, 65, 66, 71–73, 76, 77, 79, 81–83, 146–148, 150–152], *screening* (*n* = 51/82) [8, 17, 29, 40, 41, 45, 53, 57, 58, 60, 62, 71–73, 76, 79, 83, 84, 86–89, 92–95, 97, 98, 102, 113, 116, 132–145, 147, 148, 150–153], *selection* (*n* = 20/82) [8, 29, 40, 41, 53, 57, 71–73, 76, 79, 101–103, 134, 138, 139, 148, 151, 152], *extraction* (*n* = 17/82) [8, 17, 29, 40, 41, 45, 58, 71, 79, 108, 110, 114, 139, 148, 150, 154, 155], *critical quality appraisal* (*n* = 9/82) [8, 29, 40, 41, 57, 66, 119, 121, 147] and *synthesis* (*n* = 17/82) [17, 29, 40, 41, 45, 53, 57, 58, 71, 124–128, 147, 150, 151], as detailed in Table 2.

### Access costs for HICs and LMICs

The majority of studies included (*n* = 100/137; 73%) did not report cost for accessing the AI tools and/or platforms across all countries. Only 27% (*n* = 37/137) did report, as described in **Supplementary Table S4**. The majority of countries identified (*n* = 34) as either developing and/or (also) implementing AI tools and platforms to optimise any or all of the ES steps, which also reported access costs, were HICs. As such, we outline that 51.6% (*n* = 200/388) of such AI tools and platforms did not report cost information, 31.4% (*n* = 122/388) were open source/free for access, while 14.9% (*n* = 58/388) were proprietary, and only 2.1% (*n* = 8/388) offered differential access for a demo version (fewer steps) and/or a restricted period trial prior to subscription (payment for access) to both HICs and/or LMICs alike.

## Discussion

Overall, we identified 388 AI tools and platforms optimising any ES step from the 137 publications included but especially for study searching, screening/selection, extraction and summary/synthesis. For optimisation by LLMs, we observed that this was mainly for data extraction and synthesis, and that these have mostly been developed and implemented recently (since 2020).

Compared to other ES exercises to identify AI tools and platforms [17, 29–31, 38, 39, 41, 42, 46, 49, 61, 67, 130, 131, 148, 153, 156, 157], including those that are permanently being updated [44, 149], our scoping review is the first to identify and map all data science methods and include tools that are currently being either developed and/or implemented across LMICs and HICs to optimise all ES steps, including LLMs.

A key finding from our review is the evidence gap from African, Latin American and Middle East LMICs, which further outlines not only the lack of funding for such types of research and development in these geographical areas but also of the implementation of such AI tools and platforms — a crucial inequity issue if we consider inclusiveness and the wider societal impact of ES used to inform decisions in both LMICs and HICs. In this sense, glocal capacity-building initiatives for multi-professional teams should be prioritised, as supported by PAHO [158], WHO [21, 33] and academic experts from multidisciplinary fields [22, 23, 31, 49, 130, 159–162]. As part of our review, we identified various barriers to implementing AI tools and LLMs platforms in ES that are likely to be contributing to such inequities between LMICs and HICs, including access issues due to subscription models and licensing fees, especially in LMICs. Suggestions for overcoming these barriers are detailed in our Supplementary Discussion to enhance the practical impact of our review, by including open-source AI tools and platforms, fostering multidisciplinary capacity-building initiatives and ensuring equitable access to these technologies across different regions.

### Barriers and facilitators to AI tools and platforms development and implementation

Development and implementation specialists have reported a series of concerns regarding barriers for when deploying AI tools and platforms to optimise *all* ES steps (as described in the Supplementary Discussion). Their main concern relates to *access* as, despite 1-month (extendible) trials and volume discounts for ‘site licences’, pay per use offered via subscription remains a barrier to all LMICs and some researchers’ and implementers’ groups even in HICs, even though it has been adopted by the National Institute for Health and Care Excellence (NICE) as its core ES platform, and free to use for Cochrane and Campbell reviews [40].

When it comes to facilitators for globally used subscriptions’ AI tools and platforms such as Covidence, EPPI-Reviewer version 5 was reported to be released as an open-source software to foster collaborative efforts — as a cloud-based service that supports data sharing and re-use, aiming to reduce the considerable duplication of effort across the global systematic review community. This is part of the EPPI-Centre’s goal to support ‘Living’ ES and guidelines through the ‘surveillance’ of new research evidence as they are published [40], beyond the scope of peer-reviewed journals.

Another key barrier relates to ‘Living’ ES (i.e. frequently updated manually or via automation). Schmidt and colleagues [41] have identified several ways to make ‘Living’ ES more feasible, given that most (but not all) focus on automation and efficiency of the review process for a given topic, if all processes within the workflow are sufficiently flexible to adapt to changing needs and are reported in a transparent manner. The use of dedicated staff or crowdsourcing (such as Amazon Mechanical Turk, for example), development of automated update and monitoring systems and the use of simplification and standardised methods are just some approaches that may help to accelerate processes. Pre-prints or dedicated web presence for automatic dissemination of in-progress evidence updates, rather than solely relying on peer-reviewed journal publications, is important to make the effort of undertaking ‘Living’ ES a worthwhile endeavour [41].

Nevertheless, despite offering basic ‘Living’ ES functionalities, the TM and ML RobotReviewer tool family and the four existing end-user tools (NestedKnowledge, SWIFT-ActiveScreener, DistillerSR, EPPI-Reviewer) that cover most of the workflow remain to be further developed [41]. Specialists’ main concern regards superficial and overreliance on the output from deploying such tools and platforms that could decrease scientific creativity by reducing the impact of papers in generating real contributions to a specific knowledge field [46].

Another concern lies in reviewers still preferring human–human interactions [89] and the lack of significant interest in integrating AI tools and platforms optimisation into standard SLR processes (despite how much time can be saved by their deployment) [88], especially for data extraction and critical quality appraisal steps, mainly due to research gaps in the transparent and fair comparison of the performance of such tools [48], given the most recent SLRs addressing them are old or include relatively few of the available tools due to limitations such as limited availability and access due to licensing fees [41]. Therefore, we highlight the identified preference for human–human interactions over integrating AI tools in standard systematic review processes, particularly for data extraction and quality appraisal. The importance of semi-automation and proposing ways to effectively balance human and machine inputs, including fostering transparency in AI tool outputs and maintaining human oversight in critical review steps, strengthens our work’s recommendations.

Finally, when it comes to LLMs, field-specific platforms — such as Elicit or ResearchRabbit — can identify references for a specific topic that might be missed by conventional literature searches [46]. However, in research examining widely available and deployed LLM tools such as OpenAI’s ChatGPT, its application in medical scholarship shows that the language model exhibits a disconcerting tendency to produce plausible yet erroneous content (i.e. hallucinations), including spurious references, thereby jeopardising scientific integrity and the dissemination of accurate information [49]. Therefore, improving the technical evaluation parameters of data quality's (in terms of volume [163] and validation) [98], especially sensitivity (recall), not only in widely implemented ML tools and platforms – such as AbstrackR, RobotAnalyst [142, 143], DistillerSR [164], RCT Tagger, RobotReviewer, RobotSearch, SWIFT-Active Screener, SWIFT-Review and SRA-Helper [144] but also in LLMs – such as ChatGPT [97] to avoid hallucinations by poor zero-shot performance, and reduce human workload and time, as opposed to promising new findings by including few-shot prompting for Bio-SIEVE [97] remain key.

Although ChatGPT represents a significant leap forward in NLP, employing a large scale, pre-trained neural network to generate human-like responses to user queries [96] — since it was not intended for clinical applications by design, specialists conclude that specialised NLP models trained on (bio)medical datasets still represent the right direction to pursue for critical clinical applications [47]. Their main goal is avoiding ethical concerns [48] regarding accuracy, undermining clinical reasoning and the value of human expertise, perpetuating biases, promoting conspiracy theories, and demanding human oversight, as well as further challenges around traditional roles of authorship [49]. As such, since humans and machines have different capabilities, developers can reconceive both ES individual steps as well as their ordering when machines undertake them [147] to understand at which point it is ‘safe’ for the human reviewer to stop manual screening. For now, the majority of specialists still prioritise human input and human-to-human interaction to even semi-automatised ES steps [120]. Therefore, semi-automation is recommended meanwhile [157, 165], as well as glocal discussions on the ethical implications of using AI in ES, emphasising the importance of human oversight and the development of specialised NLP models trained on biomedical datasets to ensure the reliability and ethical integrity of the AI tools used in clinical applications.

### AI tools and platforms development and implementation study limitations

Only a small portion of the publications we reviewed comprised (non-)SLRs themselves (15.33% *n* = 21/137), editorial comments (9.49% *n* = 13/137) and abstracts (2.92% *n* = 4/137) or grey literature (2.92% *n* = 4/137) — which may limit or even bias our understanding of currently available AI tools and platforms’ actual clinical utility and validity to inform decision-making in health research and practice [41, 47, 49]. This is mainly because even widely available and deployed LLMs such as ChatGPT, as well as the vast majority of tools and platforms that implemented ML, DL and NNs to optimise *all* steps of standard and ‘Living’ ES, remain a relatively new topic (i.e. mainly since the mid- 2010s). Therefore, although the majority of the publications we reviewed constituted case studies (69.34%, *n* = 95/137) that quantitatively and qualitatively experimented, described and evaluated such tools and platforms, the field still requires standardised and better detailed quantitative evaluations to make their findings across different publications directly comparable.

Standardised terminology and evaluation frameworks, incorporating data science-specific evaluation metrics, are needed for not only quantitative but also qualitative comparison across different studies, which affects the direct comparability of findings from different publications [61]. As such, we need frameworks that go beyond PRISMA-DFLLM [117] to also incorporate data science field-specific evaluation analytes — e.g. median (range) workload savings (%), mean responsiveness (%) and algorithm accuracy performance statistical scores (i.e. sensitivity, specificity, precision, recall and F1) — as well as developing new and more meaningful (i.e. multi-stakeholder validated) parameters, since results might remain inherently subjective, when reported by developers themselves, if researchers do not provide reflexivity statements [61, 166].

Furthermore, precision is subjective and influenced by reviewers’ expertise which can affect their screening judgements [58, 112, 113, 142]. Therefore, error analysis technique requires further detailed elucidation and validation [83, 114, 132, 139], including specific reporting checklists for protocols and manuscripts for these types of experiments or for ES that are being optimised by AI tools and platforms [138]. It is also essential to outline which types of (hybrid) data science methods are being deployed in each tool and/or platform, as well as code availability [41] and a ‘manual arm’ to enable the relative time use estimation of AI tools and platforms against manual practices [120].

A limitation of studies developing and implementing ES *protocol planning* is no access to multilingual annotated PICO corpora for testing [149]. Therefore, wider training remains to be tested. When it comes to *study search*, *screening*, *selection* and *data extraction*, developers and implementers outlined the need for larger sample evaluation of all types of data science tools and platforms being either developed and/or implemented [51, 64, 156], as well as shortcomings related to automated database search coverage, especially regarding grey literature (i.e. Google Scholar and the OpenGrey), as well as English language limitations [31] that should be tackled by all multi-stakeholders. There was also a final recommendation to further invest in adequately improving the basic ‘Lliving’ ES functionalities as offered by open-access tools and platforms [41].

Our scoping review is not without its own limitations as, although there were no restrictions with respect to language or time, only English keywords were used in our search strategies across all databases. Therefore, publications included may not represent all the literature produced in non-English-speaking countries, and language bias may have been introduced. This has also been the case in at least one other ES included [31]. Following scoping review guidelines, we have also not critically appraised the quality of included publications, as our main goal was to identify gaps in the literature as well as the state of the art in the landscape of AI tools and platforms for ES [34]. Finally, synthesis was performed only by MSAS, but all findings and discussion have been revised by all reviewers that extracted data as well as project lead authors.

### Concluding remarks

Given the WHO’s calls for safe and ethical AI in health [33], we outline three key recommendations for developers and implementers of the landscape of AI tools and platforms for ES optimisation that we identified in this scoping review.

First, experts should avoid stand-alone tools, support more democratic AI platforms to enable more collaborative works across fields, be inclusive of the gap regions identified (Latin America, Africa, Middle East), and consider equity of access for both HICs and LMICs to AI tools and platforms that optimise the various ES steps.

Second, the field requires evaluation standards for methods testing and reporting across fields. As such, another key facilitator is developing AI tools and platforms in a way that verification and transparency are fully enabled at all levels.

Third, human input remains essential at all ES steps. Therefore, fostering multidisciplinary capacity-building opportunities and building multi-professional teams are key facilitators for both development and implementation of safe and ethical AI tools and platforms in health.

## Supplementary Information

Supplementary Table S1 – Preferred Reporting Items for Systematic Reviews and Meta-analyses (PRISMA) extension for Scoping Reviews (PRISMA-ScR) Reporting Guidelines [1]. Supplementary Table S2 – Data Extraction Form. Supplementary Table S3 – Included and Excluded Studies Reference Lists (with reasons for exclusion). Supplementary Table S4 – Global AI tools and platforms being developed and/or implemented for ES steps automation. Supplementary Findings: Regional Specialisation – Findings per Region and Country. I – Asia-Pacific. II – Europe. III – Middle-East. IV – North America. V – South America. Supplementary Discussion: Barriers and Facilitators to AI Tools and Platforms Development and Implementation. I – ES Planning and Protocol Writing. II – Search. III – Screening and Selection. IV – Reference Management and De-duplication. V – Data Extraction. VI – Critical Quality Appraisal. VII – Summary/ Synthesis, Updating and Dissemination.

## Data Availability

All data generated or analysed during this study are included in this published article (and its supplementary information files).

## Footnotes

1 OSF is a free, open-source web application that connects and supports the research workflow, enabling scientists to increase the efficiency and effectiveness of their research. OSF is the flagship product of the nonprofit Center for Open Science.
