# Supplementary material for: The landscape of artificial intelligence tools and platforms for evidence synthesis: a scoping review
Source: Syst Rev. 2026 Feb 10;15:82. doi: 10.1186/s13643-025-02842-y (PMC12998105; doi:10.1186/s13643-025-02842-y)
Supplement: Supplementary file 1 — Supplementary Table S1 – Preferred Reporting Items for Systematic Reviews and Meta-analyses (PRISMA) extension for Scoping Reviews (PRISMA-ScR) Reporting Guidelines [1]. Supplementary Table S2 – Data Extraction Form. Supplementary Table S3 – Included and Excluded Studies Reference Lists (with reasons for exclusion). Supplementary Table S4 – Global AI tools and platforms being developed and/or implemented for ES steps automation. Supplementary Findings: Regional Specialisation – Findings per Region and Country. I – Asia-Pacific. II – Europe. III – Middle-East. IV – North America. V – South America. Supplementary Discussion: Barriers and Facilitators to AI Tools and Platforms Development and Implementation. I – ES Planning and Protocol Writing. II – Search. III – Screening and Selection. IV – Reference Management and De-duplication. V – Data Extraction. VI – Critical Quality Appraisal. VII – Summary/ Synthesis, Updating and Dissemination. [file 13643_2025_2842_MOESM1_ESM.docx]

### **SUPPLEMENTARY FILES**

### **Supplementary Tables**

**Supplementary Table S1 – Preferred Reporting Items for Systematic Reviews and Meta-analyses (PRISMA) extension for Scoping Reviews (PRISMA-ScR) Reporting Guidelines** [1]**.**

| **SECTION** | **ITEM** | **PRISMA-ScR CHECKLIST ITEM** | **REPORTED ON PAGE #** |
| --- | --- | --- | --- |
| **TITLE** | | | |
| Title | 1 | Identify the report as a scoping review. | 1 |
| **ABSTRACT** | | | |
| Structured summary | 2 | Provide a structured summary that includes (as applicable): background, objectives, eligibility criteria, sources of evidence, charting methods, results, and conclusions that relate to the review questions and objectives. | 2 |
| **INTRODUCTION** | | | |
| Rationale | 3 | Describe the rationale for the review in the context of what is already known. Explain why the review questions/objectives lend themselves to a scoping review approach. | 2-4 |
| Objectives | 4 | Provide an explicit statement of the questions and objectives being addressed with reference to their key elements (e.g., population or participants, concepts, and context) or other relevant key elements used to conceptualize the review questions and/or objectives. | 4 |
| **METHODS** | | | |
| Protocol and registration | 5 | Indicate whether a review protocol exists; state if and where it can be accessed (e.g., a Web address); and if available, provide registration information, including the registration number. | 4-6 |
| Eligibility criteria | 6 | Specify characteristics of the sources of evidence used as eligibility criteria (e.g., years considered, language, and publication status), and provide a rationale. | 6 |
| Information sources* | 7 | Describe all information sources in the search (e.g., databases with dates of coverage and contact with authors to identify additional sources), as well as the date the most recent search was executed. | 5-6 |
| Search | 8 | Present the full electronic search strategy for at least 1 database, including any limits used, such that it could be repeated. | 5-6 |
| Selection of sources of evidence† | 9 | State the process for selecting sources of evidence (i.e., screening and eligibility) included in the scoping review. | 6 |
| Data charting process‡ | 10 | Describe the methods of charting data from the included sources of evidence (e.g., calibrated forms or forms that have been tested by the team before their use, and whether data charting was done independently or in duplicate) and any processes for obtaining and confirming data from investigators. | 6-7 |
| Data items | 11 | List and define all variables for which data were sought and any assumptions and simplifications made. | 6-7 |
| Critical appraisal of individual sources of evidence§ | 12 | If done, provide a rationale for conducting a critical appraisal of included sources of evidence; describe the methods used and how this information was used in any data synthesis (if appropriate). | Not performed |
| Synthesis of results | 13 | Describe the methods of handling and summarizing the data that were charted. | 6-7 |
| **RESULTS** | | | |
| Selection of sources of evidence | 14 | Give numbers of sources of evidence screened, assessed for eligibility, and included in the review, with reasons for exclusions at each stage, ideally using a flow diagram. | 7 |
| Characteristics of sources of evidence | 15 | For each source of evidence, present characteristics for which data were charted and provide the citations. | 8-9 |
| Critical appraisal within sources of evidence | 16 | If done, present data on critical appraisal of included sources of evidence (see item 12). | Not performed |
| Results of individual sources of evidence | 17 | For each included source of evidence, present the relevant data that were charted that relate to the review questions and objectives. | 9-35 |
| Synthesis of results | 18 | Summarize and/or present the charting results as they relate to the review questions and objectives. | 9-35 |
| **DISCUSSION** | | | |
| Summary of evidence | 19 | Summarize the main results (including an overview of concepts, themes, and types of evidence available), link to the review questions and objectives, and consider the relevance to key groups. | 35-44 |
| Limitations | 20 | Discuss the limitations of the scoping review process. | 44-45 |
| Conclusions | 21 | Provide a general interpretation of the results with respect to the review questions and objectives, as well as potential implications and/or next steps. | 45-46 |
| **FUNDING** | | | |
| Funding | 22 | Describe sources of funding for the included sources of evidence, as well as sources of funding for the scoping review. Describe the role of the funders of the scoping review. | 1-2 |

JBI = Joanna Briggs Institute; PRISMA-ScR = Preferred Reporting Items for Systematic reviews and Meta-Analyses extension for Scoping Reviews.

* Where *sources of evidence* (see second footnote) are compiled from, such as bibliographic databases, social media platforms, and Web sites.

† A more inclusive/heterogeneous term used to account for the different types of evidence or data sources (e.g., quantitative and/or qualitative research, expert opinion, and policy documents) that may be eligible in a scoping review as opposed to only studies. This is not to be confused with *information sources* (see first footnote).

‡ The frameworks by Arksey and O’Malley [2] and Levac and colleagues [2] and the JBI guidance [3, 4] refer to the process of data extraction in a scoping review as data charting*.*

§ The process of systematically examining research evidence to assess its validity, results, and relevance before using it to inform a decision. This term is used for items 12 and 19 instead of "risk of bias" (which is more applicable to systematic reviews of interventions) to include and acknowledge the various sources of evidence that may be used in a scoping review (e.g., quantitative and/or qualitative research, expert opinion, and policy document).

**Supplementary Table S2 – Data Extraction Form.**

<https://osf.io/ayb2r/?view_only=b682b48a91d0497e8451bf6cce36edd5>

**Supplementary Table S3 – Included and Excluded Studies Reference Lists (with reasons for exclusion).**

<https://osf.io/ayb2r/?view_only=b682b48a91d0497e8451bf6cce36edd5>

**Supplementary Table S4 – Global AI tools and platforms being developed and/or implemented for ES steps automation.**

| **ES Step(s)** | | **Tool/Platform**  **(*developing at Implementation Stage)** | **Data Science Method(s)** | **Access Cost** | **Link** |
| --- | --- | --- | --- | --- | --- |
| **All** | | • CADIMA* | N/A | free | <https://www.cadima.info/> |
|  |  | • ChatGPT* | LLM | free/$ | <https://openai.com/chatgpt> |
|  |  | • Covidence* | ML | $ | <https://www.covidence.org/> |
|  |  | • Colandr* | NLP, SupervisedML | free | [https://www.colandrapp.com](https://www.colandrapp.com/) |
|  |  | • DistillerSR* | NLP, SupervisedML | $ | <https://www.distillersr.com/> |
|  |  | • Elicit | LLM | $ | [www.elicit.org](http://www.elicit.org/) |
|  |  | • EPPI-Reviewer* | ML, NLP | $ | <https://eppi.ioe.ac.uk/eppireviewer-web> |
|  |  | • Giotto Compliance* | ML | $ | <https://www.compliance.giotto.ai/> |
|  |  | • JBI SUMARI* [Joanna Briggs Institute System for the Unified Management of the Assessment and Review of Information] | N/A | $ | <https://sumari.jbi.global/> |
|  |  | • LitStream* | N/A | N/A | <https://www.icf.com/work/research-evaluation/litstream-systematic-literature-review> |
|  |  | • NestedKnowledge* | ML | free | <https://nested-knowledge.com/> |
|  |  | • PICO Portal* | NLP, SemiSupervisedML | $ | [https://picoportal.org](https://picoportal.org/) |
|  |  | • Scispace Copilot | LLM | $ | <https://typeset.io/> |
|  |  | • ResearchRabbit | LLM | free | <https://www.researchrabbit.ai/> |
|  |  | • RevMan* | N/A | $ | <https://training.cochrane.org/online-learning/core-software/revman> |
|  |  | • SRDB.PRO | N/A | $/free for academic use | <https://www.srdb.pro/srdbpro> |
|  |  | • SRDR+* [Systematic Review Data Repository] | N/A | N/A | <https://srdrplus.ahrq.gov/> |
|  |  | • SRToolbox | N/A | N/A | <http://systematicreviewtools.com/> |
|  |  | • SyRF* | NLP | free | [http://syrf.org.uk](http://syrf.org.uk/) |
|  |  | • Sysrev* | SupervisedML | $ | [https://sysrev.com](https://sysrev.com/) |
| **Identify the issue and determine the question** | **Planning** | • Cochrane TaskExchange* | N/A | free | <https://epoc.cochrane.org/news/task-exchange> |
|  |  | • Cochrane Crowd* | N/A | free | <https://crowd.cochrane.org/> |
|  |  | • CrowdCARE* | N/A | free | <https://crowdcare.unimelb.edu.au/index.html?g=true&ts=1666711451071&page=> |
|  |  | • PubMed PICO Tool* | N/A | free | <https://pubmedhh.nlm.nih.gov/pico/index.php> |
|  |  | • PredicTER* | N/A | N/A | <https://doi.org/10.1186/s12874-022-01805-4> |
|  |  | • COVID-SEE* | N/A | N/A | <https://doi.org/10.1016/j.xcrm.2022.100860> |
| **Write a protocol** | | • BERT* [Bidirectional Encoder Representations from Transformers architecture] | NN, LLM | N/A | <https://doi.org/10.12688/f1000research.51117.2> |
|  |  | • BioIngine* [Q-UEL (Quantum Universal Exchange Language) of XML-like tags] | DM | $ | <https://www.bioingine.com/> |
|  |  | • JBI SUMARI* [System for the Unified Management of the Assessment and Review of Information] | N/A | $ | <https://sumari.jbi.global/> |
|  |  | • Methods Wizard* | N/A | N/A | <https://doi.org/10.1016/j.xcrm.2022.100860> |
|  |  | • PredicTER* | N/A | N/A | <https://doi.org/10.1186/s12874-022-01805-4> |
|  |  | • PubMed PICO* | TM | free | pubmedhh.nlm.nih.gov/nlmd/pico/piconew.php |
|  |  | • QA-BERT* [question-answering model that predicts the locations of PICO entities within sentences] | NN, LLM | N/A | <https://arxiv.org/abs/2001.11268> |
|  |  | • SRToolbox | N/A | N/A | <http://systematicreviewtools.com/> |
|  |  | • SciBERT* [model based on the BERT-base architecture, with further pre-trained weights based ontexts from the Semantic Scholar search engine] | NN, LLM | N/A | <https://github.com/allenai/scibert/> |
|  |  | • Swift-Review* | SupervisedML | free | <https://www.sciome.com/swift-review> |
|  |  | • Template* | N/A | N/A | <https://doi.org/10.1016/j.xcrm.2022.100860> |
|  |  | • Trialstreamer* | NLP, SupervisedML | free | <https://trialstreamer.ieai.robotreviewer.net/> |
| **Search for studies** | **Search** | • 2DSearch* | NLP | $ | [https://www.2dsearch.com](https://www.2dsearch.com/) |
|  |  | • A2A | N/A | N/A | <https://doi.org/10.1186/s12874-022-01805-4> |
|  |  | • AcroMine | TM | free | <https://www.nactem.ac.uk/software/termine/> |
|  |  | • Active PubMed Search [APS] | ML | N/A | <https://doi.org/10.1016/j.jbi.2023.104389> |
|  |  | • Aggregator | SupervisedML | N/A | <http://arrowsmith.psych.uic.edu/cgi-bin/arrowsmith_uic/RCT_Tagger.cgi?ID=22379> |
|  |  | • Ananse | NLP, Keyword CoOccurrance Network | free | <https://github.com/baasare/ananse> |
|  |  | • Anne O'Tate | TM | free | <http://arrowsmith.psych.uic.edu/cgi-bin/arrowsmith_uic/AnneOTate.cgi> |
|  |  | • APSE | N/A | N/A | <https://doi.org/10.1186/s12874-022-01805-4> |
|  |  | • ASSERT* [Automatic Summarisation for Systematic Reviews using Text Mining] Project Tools | TM | free | <https://www.nactem.ac.uk/assert/> |
|  |  | • askMEDLINE* | TM, NLP | free | askmedline.nlm.nih.gov/ask/ask.php |
|  |  | • BADERI | N/A | N/A | <https://doi.org/10.1186/s12874-022-01805-4> |
|  |  | • BEST | N/A | N/A | <https://doi.org/10.1186/s12874-022-01805-4> |
|  |  | • Bibliography Bot [BIBOT]* | NLP, MLAlgorithm | free | <https://github.com/Nurtal/BIBOT-light-version> |
|  |  | • BiblioShiny* | TM | free | <https://www.bibliometrix.org/home/index.php/layout/biblioshiny> |
|  |  | • Bioreader | N/A | N/A | <https://doi.org/10.1186/s12874-022-01805-4> |
|  |  | • Best-evidence Retrieval and Delivery (BiRD) system | NLP | N/A | <https://doi.org/10.1177/1460458205050684> |
|  |  | • (Brassey et al., 2021) Tool | ML, NLP | free | <https://doi.org/10.1136/bmjebm-2018-111126> |
|  |  | • Carrot2* | NLP | free | [https://search.carrot2.org](https://search.carrot2.org/) |
|  |  | • CDSS-KB [Clinical Decision Support System Knowledge Base] | HL7 Arden Syntax, Medical Logic Module, KnowledgeButton, TM | N/A | <https://doi.org/10.3390/s150921294> |
|  |  | • ChatGPT [based on GPT-3.5] | LLM | free/$ | <https://openai.com/chatgpt> |
|  |  | • Chilibot* | NLP | free | [http://www.chilibot.net](http://www.chilibot.net/) |
|  |  | • Citationchaser | N/A | N/A | <https://doi.org/10.1186/s12874-022-01805-4> |
|  |  | • CitNetExplorer* | TM | free | <https://www.citnetexplorer.nl/> |
|  |  | • CloudSERA | N/A | N/A | <https://doi.org/10.1186/s12874-022-01805-4> |
|  |  | • Cochrane RCT Classifier* [Screen4Me] | ML | free | <https://training.cochrane.org/online-learning/good-practice-resources-cochrane-authors/screen4me> |
|  |  | • Cochrane Register of Studies* | NLP | N/A | <https://community.cochrane.org/help/tools-and-software/crs-cochrane-register-studies> |
|  |  | • Colandr* | NLP, SupervisedML | free | [https://www.colandrapp.com](https://www.colandrapp.com/) |
|  |  | • CORD-19* [Okapi BM25, BioBERT, GenesisAI] | TM, ML | free/$ | <https://doi.org/10.1016/j.jclinepi.2022.04.027> |
|  |  | • COREMINE medical* | NLP | N/A | <http://www.coremine.com/medical> |
|  |  | • Costumer* | SupervisedML | free | <https://github.com/UBESP-DCTV/costumer> |
|  |  | • CTD | N/A | N/A | <https://doi.org/10.1186/s12874-022-01805-4> |
|  |  | • DASyR | TM, SupervisedML, DecisionTreeClassifiers | N/A | not identified |
|  |  | • DBPedia | TM, MLAlgorithm | free | <https://www.dbpedia.org/resources/live/dbpedia-live-sync/> |
|  |  | • (Denzler et al., 2021) Artifact | ML, TextAnalytics | N/A | <https://aisel.aisnet.org/amcis2021/art_intel_sem_tech_intelligent_systems/art_intel_sem_tech_intelligent_systems/4> |
|  |  | • DistillerSR* | NLP, SupervisedML | $ | <https://www.distillersr.com/> |
|  |  | • DOC Search | N/A | N/A | <https://doi.org/10.1186/s12874-022-01805-4> |
|  |  | • Doctor Evidence* | NLP | $ | [https://www.drevidence.com](https://www.drevidence.com/) |
|  |  | • Epistemonikos* | SupervisedML | N/A | [https://www.epistemonikos.org](https://www.epistemonikos.org/) |
|  |  | • EPPI-Reviewer* | ML | $ | <https://eppi.ioe.ac.uk/EPPIReviewer-Web/home> |
|  |  | • FACTA+* | NLP | N/A | <http://www.nactem.ac.uk/facta> |
|  |  | • Federated Search tool | TM | N/A | <https://doi.org/10.1109/APSEC.2017.10> |
|  |  | • Findpapers | N/A | N/A | <https://doi.org/10.1186/s12874-022-01805-4> |
|  |  | • Google Translate | N/A | free | <https://translate.google.com/> |
|  |  | • GScraper | N/A | N/A | <https://doi.org/10.1186/s12874-022-01805-4> |
|  |  | • Health Database Advanced Search [HDAS by NICE] | ML | N/A | <http://dx.doi.org/10.1016/j.jclinepi.2017.08.011> |
|  |  | • Heoro* | NLP | N/A | [https://www.heoro.com](https://www.heoro.com/) |
|  |  | • IBM PARSe* | TM, ML | free | <https://www.ibm.com/docs/en/api-connect/10.0.1.x?topic=execute-parse> |
|  |  | • Import.io | N/A | N/A | <https://doi.org/10.1186/s12874-022-01805-4> |
|  |  | • Inciteful.xyz | N/A | N/A | <https://doi.org/10.1186/s12874-022-01805-4> |
|  |  | • IRIS.AI* | NLP, SupervisedML | $ | [https://the.iris.ai](https://the.iris.ai/) |
|  |  | • Jane | N/A | N/A | <https://doi.org/10.1186/s12874-022-01805-4> |
|  |  | • JBI SUMARI* [Joanna Briggs Institute System for the Unified Management of the Assessment and Review of Information] | N/A | $ | <https://sumari.jbi.global/> |
|  |  | • JSTOR Text Analyzer* | TM | $ | <https://southern.libguides.com/c.php?g=1129860&p=8245790> |
|  |  | • Keyword Analyzer* | TM | $ | <https://www.elastic.co/guide/en/elasticsearch/reference/current/analysis-keyword-analyzer.html> |
|  |  | • LDS Shiny | N/A | N/A | <https://doi.org/10.1186/s12874-022-01805-4> |
|  |  | • Leaf | N/A | N/A | <https://doi.org/10.1186/s12874-022-01805-4> |
|  |  | • Leximancer* | NLP, UnsupervisedML | $ | [https://www.leximancer.com](https://www.leximancer.com/) |
|  |  | • Lingo3G3* | UnsupervisedML | $ | <https://carrotsearch.com/lingo3g/> |
|  |  | • Lingo4G* | TM | $ | <https://carrotsearch.com/lingo4g/> |
|  |  | • Linguamatics* | NLP | $ | [https://www.linguamatics.com](https://www.linguamatics.com/) |
|  |  | • LiSA* [Literature Search Application] | DL, NER, LLM (BERT) | N/A | <https://doi.org/10.1186/s12911-022-02085-0> |
|  |  | • Litbaskets | N/A | N/A | <https://doi.org/10.1109/ICAPAI55158.2022.9801564> |
|  |  | • LiteRev* | NLP, UnsupervisedML | free but limited to open-access databases with free APIs to abstract or full-text papers | <https://literev.unige.ch/> |
|  |  | • Litmaps | N/A | N/A | <https://doi.org/10.1186/s12874-022-01805-4> |
|  |  | • Litsearchr* | TM, NLP | free | <https://github.com/elizagrames/litsearchr> |
|  |  | • LitSonar | N/A | N/A | <https://doi.org/10.1177/02683962211048201> |
|  |  | • LitSuggest* | MLAlgorithm | free | <https://www.ncbi.nlm.nih.gov/research/litsuggest/> |
|  |  | • Mapping MEDLINE | N/A | N/A | <https://doi.org/10.1186/s12874-022-01805-4> |
|  |  | • Medline (Pubmed) trend | N/A | N/A | <https://doi.org/10.1186/s12874-022-01805-4> |
|  |  | • Medline Transpose | N/A | N/A | <https://doi.org/10.1186/s12874-022-01805-4> |
|  |  | • MeSHonDemand* | TM | free | <https://www.nlm.nih.gov/oet/ed/mesh/meshondemand.html> |
|  |  | • MeSHSIM | N/A | N/A | <https://doi.org/10.1186/s12874-022-01805-4> |
|  |  | • MetaPreg* | ML, NLP | N/A | <http://metapreg.org/> |
|  |  | • MetaSearcher model | TM | N/A | not identified |
|  |  | • Metta | TM, Federated search model | $ | <http://mengs1.cs.binghamton.edu/metta/search.action> |
|  |  | • MiSearch | ML | N/A | <https://doi.org/10.1016/j.ymeth.2015.01.015> |
|  |  | • (Ruiz et al., 2021) MTTR [Method and Tool for Generating Table of Relevance] | TM | free | <https://repository.londonmet.ac.uk/id/eprint/6931> |
|  |  | • NAILS | N/A | N/A | <https://doi.org/10.1186/s12874-022-01805-4> |
|  |  | • NestedKnowledge* | N/A | free | <https://nested-knowledge.com/> |
|  |  | • OmixLitMiner | N/A | N/A | <https://doi.org/10.1186/s12874-022-01805-4> |
|  |  | • Paperscraper | N/A | N/A | <https://doi.org/10.1186/s12874-022-01805-4> |
|  |  | • ParsCit | N/A | N/A | <https://doi.org/10.1016/j.jclinepi.2021.12.005> |
|  |  | • PDQ-Evidence | N/A | N/A | <https://doi.org/10.1186/s12874-022-01805-4> |
|  |  | • (Pérez-Pérez et al., 2022) Approach [OSCAR4, open-source chemistry analysis routines; TMCHEM, open-source alternative for identifying chemical names; DNORM, recognizes and normalizes disease names; in-house ontology-based NER] | UnsupervisedTM, SemiAutomaticML, NamedEntityRecognizers, Ontology | free | <https://doi.org/10.1016/j.neucom.2021.10.100> |
|  |  | • Pex | VisualTM | N/A | <https://doi.org/10.1186/s12874-022-01805-4> |
|  |  | • PubMed PICO* | TM | free | pubmedhh.nlm.nih.gov/nlmd/pico/piconew.php |
|  |  | • Polyglot Search Translator | N/A | N/A | <https://doi.org/10.1016/j.jclinepi.2021.12.005> |
|  |  | • Pythia | NLP, DL, ML | N/A | <https://doi.org/10.1016/j.jclinepi.2022.06.007> |
|  |  | • Publish or Perish | N/A | N/A | <https://doi.org/10.1186/s12874-022-01805-4> |
|  |  | • PubCrawler* | TM | free | pubcrawler.gen.tcd.ie/ |
|  |  | • PubGet [RightFind]* | TM | free | pubget.com/ |
|  |  | • PubReMiner* | TM | free | hgserver2.amc.nl/cgi-bin/miner/miner2.cgi |
|  |  | • PubVenn | N/A | N/A | <https://doi.org/10.1186/s12874-022-01805-4> |
|  |  | • Quick Clinical [Federated meta-search engine] | N/A | N/A | <https://doi.org/10.1186/2046-4053-3-74> |
|  |  | • RAx [enagoRead]* | NLP, SupervisedML | $ | [https://raxter.io](https://raxter.io/) |
|  |  | • RCT tagger* [LibSVM classifier] | NLP, SupervisedML | N/A | <http://arrowsmith.psych.uic.edu/cgi-bin/arrowsmith_uic/RCT_Tagger.cgi> |
|  |  | • Reflective Random Indexing [RRI] | TM | N/A | <https://doi.org/10.1016/j.jbi.2010.04.001> |
|  |  | • ReLis* | N/A | free | <https://relis.iro.umontreal.ca/auth.html> |
|  |  | • Researchr | N/A | N/A | <https://doi.org/10.1186/s12874-022-01805-4> |
|  |  | • Revtools [R package]* | NLP | free | <https://cran.rproject.org/package=revtools> |
|  |  | • RoBERTa | DL, LLM (BERT-likeTransformer) | N/A | [https://doi.org/10.1007/s40264-023-01367-4](https://doi.org/10.1007/s40264-023-01367-4https://doi.org/10.12688/f1000research.51117.2)  [https://doi.org/10.12688/f1000research.51117.2](https://doi.org/10.1007/s40264-023-01367-4https://doi.org/10.12688/f1000research.51117.2) |
|  |  | • RobotAnalyst* | SupervisedML | N/A | <http://www.nactem.ac.uk/robotanalyst> |
|  |  | • RobotSearch* | ML | $ | <http://www.robotsearch.com/> |
|  |  | • Sample Size SearchTool for PubMed* | NLP | N/A | [https://ihealth.uemc.es](https://ihealth.uemc.es/) |
|  |  | • Sci2 Tool | N/A | N/A | <https://doi.org/10.1186/s12874-022-01805-4> |
|  |  | • Scientific Evidence Explorer | N/A | N/A | <https://doi.org/10.1016/j.xcrm.2022.100860> |
|  |  | • Search Builder 1.0* | DM | free | <http://dx.doi.org/10.1017/S0266462315000136> |
|  |  | • SearchRefiner | N/A | N/A | <https://doi.org/10.1186/s12874-022-01805-4> |
|  |  | • SensPrecOptimizer | N/A | N/A | <https://doi.org/10.1186/s12874-022-01805-4> |
|  |  | • Sherlock | N/A | N/A | <https://doi.org/10.1016/j.jclinepi.2021.12.005> |
|  |  | • SLRqub | N/A | N/A | <https://doi.org/10.1186/s12874-022-01805-4> |
|  |  | • SLR_SearchStrings | NLP, MLAlgorithm | free | <http://bit.ly/2PwL37v> |
|  |  | • Systematic Online Living Evidence Summaries [SOLES] | N/A | free | <https://doi.org/10.1042/CS20220494> |
|  |  | • SRA [Systematic Review Accelerator]* | NLP | free | [https://sr-accelerator.com](https://sr-accelerator.com/) |
|  |  | • SRToolbox | N/A | N/A | <http://systematicreviewtools.com/> |
|  |  | • (Surian et al., 2018) Matrix Factorisation Approach | ML | N/A | <https://doi.org/10.1016/j.jbi.2018.01.008> |
|  |  | • SWIFT ActiveScreener* | ML | $ | <https://www.sciome.com/swift-activescreener/> |
|  |  | • Swift-Review* | TM, SupervisedML | free | <https://www.sciome.com/swift-review> |
|  |  | • TerMine* | TM | free | <https://www.nactem.ac.uk/software/termine/> |
|  |  | • Textpresso | N/A | N/A | <https://doi.org/10.1186/s12874-022-01805-4> |
|  |  | • TextRank | NLP, UnsupervisedML | free | <https://cran.r-project.org/web/packages/textrank/index.html> |
|  |  | • Thalia* | NLP | N/A | <http://nactem-copious.man.ac.uk/Thalia> |
|  |  | • TheoryOn | N/A | N/A | [https://doi.org/10.1109/ICAPAI55158.2022.9801564](https://doi.org/10.1109/ICAPAI55158.2022.9801564https://doi.org/10.1177/02683962211048201)  [https://doi.org/10.1177/02683962211048201](https://doi.org/10.1109/ICAPAI55158.2022.9801564https://doi.org/10.1177/02683962211048201) |
|  |  | • (Timsina et al., 2015) Unnamed Approach | ML | N/A | <https://core.ac.uk/download/pdf/301365687.pdf> |
|  |  | • Tm for R | TM | free | <https://cran.r-project.org/web/packages/tm/index.html> |
|  |  | • Trial2rev | SemiSupervisedML | free | <https://github.com/evidence-surveillance/trial2rev> |
|  |  | • Trialstreamer* | DM, NLP, SupervisedML | free | <https://trialstreamer.ieai.robotreviewer.net/> |
|  |  | • Twister [Text wizard for semi-automated literature reviews] | TM | N/A | [https://doi.org/10.1016/j.jbi.2023.104389](https://doi.org/10.1016/j.jbi.2023.104389https://doi.org/10.3895/rts.v16n45.12119)  [https://doi.org/10.3895/rts.v16n45.12119](https://doi.org/10.1016/j.jbi.2023.104389https://doi.org/10.3895/rts.v16n45.12119) |
|  |  | • Unified Medical Language System [UMLS] Metathesaurus* | TM | N/A | [https://doi.org/10.1016/j.jbi.2011.05.007](https://doi.org/10.1016/j.jbi.2011.05.007https://doi.org/10.1016/j.artmed.2006.07.012)  [https://doi.org/10.1016/j.artmed.2006.07.012](https://doi.org/10.1016/j.jbi.2011.05.007https://doi.org/10.1016/j.artmed.2006.07.012) |
|  |  | • VOS [Visualisation of Similarities] Viewer* | TM | N/A | [https://doi.org/10.1186/s12874-022-01805-4](https://doi.org/10.1186/s12874-022-01805-4https://doi.org/10.1002/jrsm.1665)  [https://doi.org/10.1002/jrsm.1665](https://doi.org/10.1186/s12874-022-01805-4https://doi.org/10.1002/jrsm.1665) |
|  |  | • Voyant Tools* | NLP | free | [https://voyant-tools.org](https://voyant-tools.org/) |
|  |  | • XplorMed* | NLP | N/A | <http://www.bork.emblheidelberg.de/xplormed/> |
|  |  | • Weka | TM, ML | $ | <https://www.weka.io/> |
|  |  | • Wordstat 9.0* | NLP | $ | <https://provalisresearch.com/products/content-analysis-software> |
|  |  | • Yale MeSH Analyser* | TM | free | <https://mesh.med.yale.edu/> |
|  | **Reference Management** | • BibDesk | N/A | free | <https://bibdesk.sourceforge.io/> |
|  |  | • Citavi | N/A | $ | <https://lumivero.com/products/citavi/> |
|  |  | • Colwiz | N/A | free | <https://colwiz.findmysoft.com/> |
|  |  | • Docear | N/A | free | <https://docear.org/> |
|  |  | • EndNote* | TM, ML | $ | <https://endnote.com/> |
|  |  | • JabRef | N/A | free | <https://www.jabref.org/> |
|  |  | • JBI SUMARI* [Joanna Briggs Institute System for the Unified Management of the Assessment and Review of Information] | N/A | $ | <https://sumari.jbi.global/> |
|  |  | • KBibTex | N/A | N/A | <https://apps.kde.org/pt-br/kbibtex/> |
|  |  | • Mendeley* | N/A | free | <https://www.mendeley.com/> |
|  |  | • Nvivo* | TM | $ | <https://lumivero.com/products/nvivo/> |
|  |  | • Paperpile | N/A | $ | <https://paperpile.com/> |
|  |  | • Papers | N/A | $ | <https://www.papersapp.com/> |
|  |  | • Qiqqa | N/A | free | <https://qiqqa.en.softonic.com/> |
|  |  | • Refbase | N/A | N/A | <https://wiki.refbase.net/Main_Page> |
|  |  | • Wikindx | N/A | free | <https://wikindx.sourceforge.io/web/trunk/index.html> |
|  |  | • Zotero* | N/A | free | <https://www.zotero.org/> |
| **Sift and select studies** | **Deduplication** | • CADIMA* | N/A | free | <https://www.cadima.info/> |
|  |  | • (Denzler et al., 2021) Artifact | ML, TextAnalytics | N/A | <https://aisel.aisnet.org/amcis2021/art_intel_sem_tech_intelligent_systems/art_intel_sem_tech_intelligent_systems/4> |
|  |  | • DistillerSR* | NLP, SupervisedML | $ | <https://www.distillersr.com/> |
|  |  | • EndNote | TM, ML | $ | <https://endnote.com/> |
|  |  | • JBI SUMARI* [Joanna Briggs Institute System for the Unified Management of the Assessment and Review of Information] | N/A | $ | <https://sumari.jbi.global/> |
|  |  | • Litsearchr* | TM, NLP | free | <https://github.com/elizagrames/litsearchr> |
|  |  | • PICO Portal* | NLP, SemiSupervisedML | $ | [https://picoportal.org](https://picoportal.org/) |
|  |  | • ProCite | N/A | N/A | <https://doi.org/10.1186/2046-4053-3-74> |
|  |  | • ReLis* | N/A | free | <https://relis.iro.umontreal.ca/user.html> |
|  |  | • Revtools [R package]* (NLP, free, ) | NLP | free | <https://cran.rproject.org/package=revtools> |
|  |  | • Systematic Online Living Evidence Summaries [SOLES] ASySD [Automated Systematic Search Deduplication tool] | N/A | free | <https://doi.org/10.1042/CS20220494> |
|  |  | • SRA [Systematic Review Accelerator] de-duplicator* | NLP | free | <https://sr-accelerator.com/#/deduplicator> |
|  | **Screening (abstract and full-text)** | • Abstrackr* | SupervisedML | free | [http://abstrackr.cebm.brown.edu](http://abstrackr.cebm.brown.edu/) |
|  |  | • Active_learning_document_screening* | NLP, SupervisedML | free | <https://github.com/afcarvallo/active_learning_document_screening> |
|  |  | • Active-learning-for-systematic-review* | NLP, SupervisedML | free | <https://github.com/sxzhang1201/active-learning-for-systematic-review> |
|  |  | • (Afzal et al., 2019) Framework | ML | free | <https://doi.org/10.2196/13430> |
|  |  | • ArticleNet | N/A | N/A | <https://doi.org/10.1186/s12874-022-01805-4> |
|  |  | • ASReview* | NLP_SupervisedML | free | [https://asreview.nl](https://asreview.nl/) |
|  |  | • ASReview-covid* | NLP, SupervisedML | free | <https://github.com/asreview/asreview-covid19> |
|  |  | • ASSERT* [Automatic Summarisation for Systematic Reviews using Text Mining] Project Tools | TM | free | <https://www.nactem.ac.uk/assert/> |
|  |  | • ART [Automatic Term Recognition] Tool | ML | N/A | <https://doi.org/10.1186/s13643-015-0117-0> |
|  |  | • (Bannach-Brown et al., 2019) Algorithm* | ML | N/A | <https://doi.org/10.1186/s13643-019-0942-7> |
|  |  | • Bibliography Bot [BIBOT]* | NLP, MLAlgorithm | free | <https://github.com/Nurtal/BIBOT-light-version> |
|  |  | • Biomed-Summarizer | DL, NN | N/A | <https://doi.org/10.2196/19810> |
|  |  | • BioReader | N/A | N/A | <https://doi.org/10.1186/s12874-022-01805-4> |
|  |  | • Bio-SIEVE* [Biomedical Systematic Include/Exclude reViewer with Explanations] | LLM (LLaMa, Guanaco) | free | <https://huggingface.co/Ambroser53/Bio-SIEVE> |
|  |  | • (Brassey et al., 2021) Tool | ML, NLP | free | <https://doi.org/10.1136/bmjebm-2018-111126> |
|  |  | • CADIMA* | N/A | free | <https://www.cadima.info/> |
|  |  | • ChatGPT | LLM | free/$ | <https://openai.com/chatgpt> |
|  |  | • Cochrane Crowd | N/A | free | <https://crowd.cochrane.org/> |
|  |  | • Cochrane RCT Classifier* | SupervisedML | free | <https://crsweb.cochrane.org/login.html> |
|  |  | • (Cohen et al., 2006) Approach | ML | N/A | <https://doi.org/10.1197/jamia.M1929> |
|  |  | • (Cohen et al., 2009) Approach | ML | N/A | <https://doi.org/10.1197/jamia.M3162> |
|  |  | • (Cohen et al., 2015) Approach* | ML | free | <https://arrowsmith.psych.uic.edu/cgi-bin/arrowsmith_uic/RCT_Tagger.cgi> |
|  |  | • Colandr* | NLP, SupervisedML | free | [https://www.colandrapp.com](https://www.colandrapp.com/) |
|  |  | • CORD-19* [Okapi BM25, BioBERT, GenesisAI] | TM, ML | free/$ | <https://doi.org/10.1016/j.jclinepi.2022.04.027> |
|  |  | • Covidence* | ML | $ | <https://www.covidence.org/> |
|  |  | • ChatGPT [based on GPT-3.5] | LLM | free/$ | <https://openai.com/chatgpt> |
|  |  | • Computational-Linguistic Approaches to Indexing and Retrieval of Text [CLARIT] | NLP | N/A | <https://pubmed.ncbi.nlm.nih.gov/1770839/> |
|  |  | • DAE-FF* | DeepNN, SupervisedML | free | <https://github.com/gkontonatsios/DAE-FF> |
|  |  | • DASyR | TM, SupervisedMLAlgorithm, SupportVectorMachine, DecisionTreeClassifiers | N/A | not identified |
|  |  | • (Denzler et al., 2021) Artifact | ML, TextAnalytics | N/A | <https://aisel.aisnet.org/amcis2021/art_intel_sem_tech_intelligent_systems/art_intel_sem_tech_intelligent_systems/4> |
|  |  | • (Dhrangadhariya et al., 2020) Citation Screening Tool | ML | N/A | <https://doi.org/10.3233/SHTI200171> |
|  |  | • DistillerAI | NLP | N/A | [https://doi.org/10.1016/j.jbi.2023.104389](https://doi.org/10.1016/j.jbi.2023.104389https://doi.org/10.1016/j.jclinepi.2021.12.005)  [https://doi.org/10.1016/j.jclinepi.2021.12.005](https://doi.org/10.1016/j.jbi.2023.104389https://doi.org/10.1016/j.jclinepi.2021.12.005) |
|  |  | • DistillerSR* | NLP, SupervisedML | $ | <https://www.distillersr.com/> |
|  |  | • DoCTER* [Document Classification and Topic Extraction Resource] | NLP, SemiSupervisedML | N/A | [https://www.icf-docter.com](https://www.icf-docter.com/) |
|  |  | • Doctor | N/A | N/A | <https://doi.org/10.1186/s12874-022-01805-4> |
|  |  | • Drug Discovery AI Factory KIBIT [Concept Encoder]* | NLP, SupervisedML | N/A | <https://lifescience.fronteousa.com/> |
|  |  | • EPPI-Reviewer* | ML, NLP | $ | <https://eppi.ioe.ac.uk/eppireviewer-web> |
|  |  | • eSuRFr | N/A | N/A | <https://abstracts.cochrane.org/2014-hyderabad/automatic-information-retrieval-citation-tracking-deduplication-and-full-text> |
|  |  | • Evidence Pipeline | N/A | N/A | <https://community.cochrane.org/help/tools-and-software/evidence-pipeline> |
|  |  | • ExaCT* | TM, ML | free | <https://exact.cluster.gctools.nrc.ca/ExactDemo/> |
|  |  | • FASTREAD (FAST2)* | SupervisedML | free | <https://github.com/fastread/src/tree/v1.4.0> |
|  |  | • (Felizardo et al., 2011) Visual TM Approach | VisualTM | N/A | <https://doi.org/10.1109/ESEM.2011.16> |
|  |  | • FCNB classifier [factorized version of the complement naïve Bayes classifier] | ML | N/A | <https://doi.org/10.1136/jamia.2010.004325> |
|  |  | • (Halpenny et al., 2022) Deep Dynamic Extract Software | ML | N/A | <https://doi.org/10.1016/j.jval.2019.09.2235> |
|  |  | • InclusionCriteria* | NLP | free | <https://github.com/infoqualitylab/InclusionCriteria> |
|  |  | • IRIS.AI* | NLP, SupervisedML | $ | [https://the.iris.ai](https://the.iris.ai/) |
|  |  | • JBI SUMARI* [System for the Unified Management of the Assessment and Review of Information] | N/A | $ | <https://sumari.jbi.global/> |
|  |  | • LiteRev* | NLP, UnsupervisedML | free but limited to open-access databases with free APIs to abstract or full-text papers | <https://literev.unige.ch/> |
|  |  | • Machine Learning Functions* | SupervisedML | free | <https://systematicreviewsjournal.biomedcentral.com/articles/10.1186/s13643-020-01520-5#Sec14> |
|  |  | • Medline Ranker* | N/A | free | <https://bio.tools/medline_ranker> |
|  |  | • MetaSearcher model | TM | N/A | not identified |
|  |  | • MMiDaS-AE [Multi-modal Missing Data aware Stacked Autoencoder] | ML, NLP | N/A | <https://doi.org/10.1145/3368555.3384463> |
|  |  | • Metagear | N/A | N/A | <https://doi.org/10.1186/s12874-022-01805-4> |
|  |  | • MetaMap | ML | N/A | [https://doi.org/10.1016/j.jbi.2010.04.001](https://doi.org/10.1016/j.jbi.2010.04.001https://doi.org/10.1109/APSEC.2017.10https://doi.org/10.1186/s13643-024-02592-3)  [https://doi.org/10.1109/APSEC.2017.10](https://doi.org/10.1016/j.jbi.2010.04.001https://doi.org/10.1109/APSEC.2017.10https://doi.org/10.1186/s13643-024-02592-3)  [https://doi.org/10.1186/s13643-024-02592-3](https://doi.org/10.1016/j.jbi.2010.04.001https://doi.org/10.1109/APSEC.2017.10https://doi.org/10.1186/s13643-024-02592-3) |
|  |  | • MetaPreg* | ML, NLP | N/A | <http://metapreg.org/> |
|  |  | • Multi-Channel CNN [Convolutional Neural Network] | N/A | N/A | <https://doi.org/10.48550/arXiv.2201.07534> |
|  |  | • NestedKnowledge* | ML | free | <https://nested-knowledge.com/> |
|  |  | • (Olorisade et al., 2019) Approach* | TM | N/A | <https://doi.org/10.1016/j.jbi.2019.103202> |
|  |  | • Papers-review | N/A | N/A | <https://doi.org/10.1186/s12874-022-01805-4> |
|  |  | • ParsCit | N/A | N/A | <https://doi.org/10.1016/j.jclinepi.2021.12.005> |
|  |  | • PARSIFAL* | N/A | free | <https://parsif.al/> |
|  |  | • (Pérez-Pérez et al., 2022) Approach [OSCAR4, open-source chemistry analysis routines; TMCHEM, open-source alternative for identifying chemical names; DNORM, recognizes and normalizes disease names; in-house ontology-based NER] | UnsupervisedTM, SemiAutomaticML, NamedEntityRecognizers, Ontology | free | <https://doi.org/10.1016/j.neucom.2021.10.100> |
|  |  | • Pex | VisualTM | N/A | <https://doi.org/10.1186/s12874-022-01805-4> |
|  |  | • (Pham et al., 2021) Unnamed Workflow | TM, NLP, ML | N/A | <https://doi.org/10.1186/s13643-021-01700-x> |
|  |  | • PICO Portal* | NLP, SemiSupervisedML | $ | [https://picoportal.org](https://picoportal.org/) |
|  |  | • Pimiento | ML | N/A | <https://doi.org/10.1109/APSEC.2017.10> |
|  |  | • PRISMA-DFLLM [Domain-specific Finetuned LLMs] | LLM | N/A | <https://doi.org/10.48550/arXiv.2306.14905> |
|  |  | • PubFinder* | TM | free | [www.glycosciences.de/ tools/PubFinder](http://www.glycosciences.de/%20tools/PubFinder) |
|  |  | • PubmedClassifier* | NLP,SupervisedML | free | <https://github.com/YujiaBao/PubmedClassifier> |
|  |  | • Pythia | NLP, DL, ML | N/A | <https://doi.org/10.1016/j.jclinepi.2022.06.007> |
|  |  | • (Queiros et al., 2022b) Tool | SupportVectorML | N/A | <https://doi.org/10.1016/j.jval.2021.11.1000> |
|  |  | • RapidMiner* | NLP, SupervisedML, SemiSupervisedML, UnsupervisedML | $ | [http://rapid-i.com](http://rapid-i.com/) |
|  |  | • RAx [enagoRead]* | NLP, SupervisedML | $ | [https://raxter.io](https://raxter.io/) |
|  |  | • Rayyan* | NLP, SupervisedML | free/$ | [https://www.rayyan.ai](https://www.rayyan.ai/) |
|  |  | • RCT Tagger* [LibSVM classifier] | NLP, SupervisedML | N/A | <http://arrowsmith.psych.uic.edu/cgi-bin/arrowsmith_uic/RCT_Tagger.cgi> |
|  |  | • ReLis* | N/A | free | <https://relis.iro.umontreal.ca/auth.html> |
|  |  | • Research Screener* | NLP, SupervisedML | free | [https://researchscreener.com](https://researchscreener.com/) |
|  |  | • ReVis | VisualTM | N/A | <https://doi.org/10.1007/s00607-023-01181-x> |
|  |  | • Revtools [R package]* | TM, NLP | free | <https://cran.rproject.org/package=revtools> |
|  |  | • RoBERTa | DL, BERT-likeTransformer | N/A | [https://doi.org/10.1007/s40264-023-01367-4 https://doi.org/10.12688/f1000research.51117.2](https://doi.org/10.1007/s40264-023-01367-4) |
|  |  | • RobotAnalyst* | SupervisedML | N/A | <http://www.nactem.ac.uk/robotanalyst> |
|  |  | • RobotReviewer/RobotSearch* | ML | $ | <http://www.robotsearch.com/> |
|  |  | • (Røst et al., 2023) NIPH [Norwegian Institute of Public Health] coding workflow | DL | N/A | <https://doi.org/10.1186/s12859-021-04396-x> |
|  |  | • (Ruiz et al., 2021) MTTR [Method and Tool for Generating Table of Relevance] | TM | free | <https://repository.londonmet.ac.uk/id/eprint/6931> |
|  |  | • Rules_cochranereviews | NLP | free | <https://github.com/dsurian/rules_cochranereviews> |
|  |  | • Screen4Me* | ML | free | <https://training.cochrane.org/online-learning/good-practice-resources-cochrane-authors/screen4me> |
|  |  | • Screen-IT | N/A | N/A | <https://doi.org/10.1186/s12874-022-01805-4> |
|  |  | • SLR_SearchStrings | NLP, MLalgorithm | free | <http://bit.ly/2PwL37v> |
|  |  | • Systematic Online Living Evidence Summaries [SOLES] | N/A | free | <https://doi.org/10.1042/CS20220494> |
|  |  | • SRA [Systematic Review Accelerator]* | NLP | free | [https://sr-accelerator.com](https://sr-accelerator.com/) |
|  |  | • SRA-Helper [for EndNote]* | N/A | free | <https://github.com/IEBH/SRA-Helper> |
|  |  | • srBERT* [Bidirectional Encoder Representations from Transformers] | LLM, ML | free | <https://github.com/SEONCHOE/> |
|  |  | • SRDB.PRO | N/A | $/free for academic use | <https://www.srdb.pro/srdbpro> |
|  |  | • SRToolbox | N/A | N/A | <http://systematicreviewtools.com/> |
|  |  | • Subscreen | N/A | N/A | <https://doi.org/10.1186/s12874-022-01805-4> |
|  |  | • State of the Art through Systematic Review [StArt]* | ML | free | <https://www.lapes.ufscar.br/resources/tools-1/start-1> |
|  |  | • (Surian et al., 2018) Matrix Factorisation Approach | ML | N/A | <https://doi.org/10.1016/j.jbi.2018.01.008> |
|  |  | • SWIFT Active Screener* | ML | $ | <https://www.sciome.com/swift-activescreener/> |
|  |  | • Swift-Review* | TM, SupervisedML | free | <https://www.sciome.com/swift-review> |
|  |  | • SyRF* | NLP | free | [http://syrf.org.uk](http://syrf.org.uk/) |
|  |  | • SYstematic Review Information Automated Collection [SYRIAC] System | ML | N/A | [https://doi.org/10.1109/APSEC.2017.10 https://doi.org/10.1016/j.jbi.2023.104389](https://doi.org/10.1016/j.jbi.2023.104389) |
|  |  | • Sys_review_ml* | ML | free | <https://github.com/toni-ml/sys_review_ml> |
|  |  | • Sysrev* | SupervisedML | $ | [https://sysrev.com](https://sysrev.com/) |
|  |  | • (Tetzlaff et al., 2019) Unnamed two parallel NLP classifiers | Naïve Bayes (NB), Support Vector Machine (SVM) | N/A | <https://doi.org/10.1016/j.jval.2019.04.1381> |
|  |  | • (Timsina et al., 2015) Unnamed Approach | ML | N/A | <https://core.ac.uk/download/pdf/301365687.pdf> |
|  |  | • (Tsafnat et al., 2018) Unnamed Approach | TM, NLP, ML, using General Architecture for Text Engineering (GATE) | N/A | <https://doi.org/10.1186/s13643-018-0724-7> |
|  |  | • (Tsubota et al., 2022) Unnamed Approach [SciBERT, SpaCy, PubMedBERT, BioELECTRA, AWS Adam optimizer, Huggingface Transformers library, Python 3.8] | LLM | N/A | <https://doi.org/10.1016/j.jbi.2022.104185> |
|  |  | • (van Altena et al., 2021) Unnamed Approach | SupervisedML | N/A | <https://doi.org/10.1002/jrsm.1518> |
|  |  | • (Xiong et al., 2018) Algorithm | ML | N/A | <https://doi.org/10.3389/fphys.2018.00835> |
|  |  | • (Wallace et al., 2012) Unnamed Approach [to Automatise Systematic Review Pipeline in Genetics] | DM, ML | free | <https://doi.org/10.1038/gim.2012.7> |
|  |  | • Weka* | ML | $ | <https://www.weka.io/> |
| **Extract data from studies** | | • A Keyword-Based Literature Review Data Generating Algorithm | NLP | free | <http://www.mdpi.com/2073-8994/12/6/903/s1> |
|  |  | • Adobe Acrobat (11) Pro DC* | TM | free/$ | <https://www.adobe.com/acrobat/acrobat-pro.html> |
|  |  | • ALBERT [A Lite BERT for Self-supervised Learning of Language Representations] | LLM | free | <https://huggingface.co/docs/transformers/en/model_doc/albert> |
|  |  | • Alexa | LLM | free | <https://developer.amazon.com/en-US/alexa/alexa-ai> |
|  |  | • (Aliyu et al, 2018) Canonical Model | TM | N/A | <https://doi.org/10.1109/SNAMS.2018.8554896> |
|  |  | • Bard* | LLM | $ | <https://blog.google/technology/ai/try-bard/> |
|  |  | • BART | LLM | free | <https://huggingface.co/docs/transformers/en/model_doc/bart> |
|  |  | • BERT [Bidirectional Encoder Representations from Transformers] | LLM | free | <https://research.google/pubs/bert-pre-training-of-deep-bidirectional-transformers-for-language-understanding/> |
|  |  | • bIOgpt | LLM | N/A | <https://huggingface.co/docs/transformers/en/model_doc/biogpt> |
|  |  | • BioMedLM* | LLM | N/A | <https://crfm.stanford.edu/2022/12/15/biomedlm.html> |
|  |  | • BLOOM(Z) | LLM | free | <https://huggingface.co/bigscience/bloomz> |
|  |  | • CERC | N/A | N/A | <https://doi.org/10.1186/s12911-020-01330-8> |
|  |  | • ChatDoctor* | LLM | $ | <https://www.chatdoctor.com/> |
|  |  | • (Chat)GLM | LLM | free | <https://github.com/THUDM/ChatGLM-6B/blob/main/README_en.md> |
|  |  | • ChatGPT* [based on GPT-3.5] | LLM | free/$ | <https://openai.com/chatgpt> |
|  |  | • Chinchilla | LLM | $ | <https://deepmind.google/> |
|  |  | • Claude* | LLM | $ | <https://claude.ai/> |
|  |  | • Clinical Camel | LLM | free | <https://doi.org/10.48550/arXiv.2305.12031> |
|  |  | • Cochrane_scraper | N/A | free | <https://github.com/DASpringate/Cochrane_scraper> |
|  |  | • CodeX* | LLM | $ | <https://openai.com/blog/openai-codex> |
|  |  | • Colandr* | NLP, SupervisedML | free | [https://www.colandrapp.com](https://www.colandrapp.com/) |
|  |  | • ContentMine | N/A | free | <https://contentmine.github.io/> |
|  |  | • Covidence* | ML | $ | <https://www.covidence.org/> |
|  |  | • Data Abstraction Assistant [DAA] | N/A | free | <https://bitbucket.org/cebmbrown/daa/src/master/> |
|  |  | • DeBERTa [Decoding-enhanced BERT with Disentangled Attention] | LLM | free | <https://huggingface.co/docs/transformers/en/model_doc/deberta-v2> |
|  |  | • Dextr | ML | N/A | <https://doi.org/10.1016/j.envint.2021.107025> |
|  |  | • DistillBERT | LLM | free | <https://huggingface.co/docs/transformers/en/model_doc/distilbert> |
|  |  | • ELECTRA | LLM | free | <https://github.com/google-research/electra> |
|  |  | • DistillerSR* | NLP, SupervisedML | $ | <https://www.distillersr.com/> |
|  |  | • ELMo [Embeddings from Language Model] | LLM | free | <https://doi.org/10.12688/f1000research.51117.2> |
|  |  | • EPPI-Reviewer* | NLP, ML | $ | <https://eppi.ioe.ac.uk/eppireviewer-web> |
|  |  | • ERNIE [Enhanced Representation through Knowledge Integration] Bot | LLM | free | <https://doi.org/10.12688/f1000research.51117.2> |
|  |  | • Evidence-based Study Extractor Tool [EvidenceSET] | N/A | N/A | <https://www.researchgate.net/publication/320193858_EvidenceSET_A_Tool_for_Supporting_Analysis_of_Evidence_and_Synthesis_of_Primary_and_Secondary_Studies#fullTextFileContent> |
|  |  | • ERNIE3.0 | LLM | $ | <https://doi.org/10.12688/f1000research.51117.2> |
|  |  | • ExaCT [EXtracting Accurate efficacy and safety information from ClinicalTrials.gov] | TM, ML | $ | <https://doi.org/10.1186/1472-6947-10-56> |
|  |  | • fastText* | LLM | free | <https://fasttext.cc/> |
|  |  | • Galactica | LLM | free | <https://gpt3demo.com/apps/meta-galactica-ai> |
|  |  | • GLaM* | LLM | $ | <https://research.google/blog/more-efficient-in-context-learning-with-glam/> |
|  |  | • Glove* [Global Vectors for Word Representation] | LLM | free | <https://nlp.stanford.edu/projects/glove/> |
|  |  | • Gopher | LLM | $ | <https://deepmind.google/> |
|  |  | • Graph2Data | N/A | N/A | <https://doi.org/10.1186/s12874-022-01805-4> |
|  |  | • GPT-1/2/GPT-Neo | LLM | free | <https://doi.org/10.12688/f1000research.51117.2> |
|  |  | • GPT-3* | LLM | $ | <https://openai.com/blog/gpt-3-apps> |
|  |  | • GPT-4* | LLM | $ | <https://openai.com/research/gpt-4> |
|  |  | • GPT-NeoX | LLM | free | <https://huggingface.co/docs/transformers/en/model_doc/gpt_neox> |
|  |  | • Import.io | N/A | N/A | <https://doi.org/10.1186/s12874-022-01805-4> |
|  |  | • InstructGPT | LLM | $ | <https://openai.com/research/instruction-following> |
|  |  | • IRIS.AI* | NLP, SupervisedML | $ | [https://the.iris.ai](https://the.iris.ai/) |
|  |  | • JBI SUMARI* [System for the Unified Management of the Assessment and Review of Information] | N/A | $ | <https://sumari.jbi.global/> |
|  |  | • Jurassic* | LLM | $ | <https://www.ai21.com/blog/announcing-ai21-studio-and-jurassic-1> |
|  |  | • Jurassic-2* | LLM | $ | <https://www.ai21.com/blog/introducing-j2> |
|  |  | • KIETA [Key-insight extraction from scientific tables] | DM | N/A | <https://doi.org/10.1007/s10489-022-03957-8> |
|  |  | • LaMDA* [Language Model for Dialogue Applications] | LLM | $ | <https://blog.google/technology/ai/lamda/> |
|  |  | • LLaMA* [Large Language Model Meta AI] | LLM | free | <https://llama.meta.com/> |
|  |  | • (Kaiser et al., 2009) LASSIE_DELTA/A | TM | N/A | <https://doi.org/10.1016/j.artmed.2008.08.009> |
|  |  | • LiSA* [Literature Search Application] | DL, NER, LLM (BERT) | N/A | <https://doi.org/10.1186/s12911-022-02085-0> |
|  |  | • MedAlpaca | LLM | N/A | <https://github.com/kbressem/medAlpaca> |
|  |  | • Med-PaLM 2* | LLM | $ | <https://sites.research.google/med-palm/> |
|  |  | • MetaDigitise | N/A | N/A | <https://cran.r-project.org/web/packages/metaDigitise/vignettes/metaDigitise.html> |
|  |  | • Metagear | N/A | free | <https://doi.org/10.1186/s12874-022-01805-4> |
|  |  | • MetaPreg* | ML, NLP | N/A | <http://metapreg.org/> |
|  |  | • Minerva* | LLM | $ | <https://research.google/blog/minerva-solving-quantitative-reasoning-problems-with-language-models/> |
|  |  | • MT-NLG | LLM | $ | <https://doi.org/10.12688/f1000research.51117.2> |
|  |  | • NaCTeM text mining tools for automatically extracting concepts relating to genes and proteins (NEMine), yeast metabolites (Yeast MetaboliNER), and anatomical entities (AnatomyTagger) | TM | N/A | <http://www.nactem.ac.uk/software.php> |
|  |  | • Named Entity Recognition [NER] | NLP, NN | N/A | <https://doi.org/10.1109/ACCESS.2021.3072900> |
|  |  | • Numbat | N/A | free | <https://github.com/bgcarlisle/Numbat> |
|  |  | • Optical Character Recognition* [OCR] | ML, DL, NN | free/$ | <https://doi.org/10.1109/ACCESS.2021.3072900> |
|  |  | • OPT [Open Pre-trained Transformer] | LLM | free | <https://huggingface.co/docs/transformers/en/model_doc/opt> |
|  |  | • PaLM* | LLM | $ | <https://ai.google/discover/palm2/> |
|  |  | • PDFBox tool* | NLP, NER, ML | N/A | <https://pdfbox.apache.org/> |
|  |  | • (Pérez-Pérez et al., 2022) Approach [OSCAR4, open-source chemistry analysis routines; TMCHEM, open-source alternative for identifying chemical names; DNORM, recognizes and normalizes disease names; in-house ontology-based NER] | UnsupervisedTM, SemiAutomaticML, NamedEntityRecognizers, Ontology | N/A | <https://doi.org/10.1016/j.neucom.2021.10.100> |
|  |  | • PICOtron | N/A | free | <https://github.com/ijmarshall/picotron> |
|  |  | • PlotDigitizer | N/A | N/A | <https://doi.org/10.1186/s12874-022-01805-4> |
|  |  | • PMC-LLaMA | LLM | N/A | <https://doi.org/10.1093/jamia/ocae045> |
|  |  | • PubMed2XL | N/A | N/A | <https://doi.org/10.1186/s12874-022-01805-4> |
|  |  | • RAPTOR | NLP | free | <https://github.com/CochraneSchizophrenia/RAPTOR> |
|  |  | • RAx [enagoRead]* | NLP, SupervisedML | $ | [https://raxter.io](https://raxter.io/) |
|  |  | • Refchaser | N/A | free | <https://github.com/DQ-Zhang/refchaser> |
|  |  | • RevMan* [Review Manager] | N/A | $ | <https://training.cochrane.org/online-learning/core-software/revman> |
|  |  | • Rnatlp | NLP | free | <https://github.com/SensorNet-UFAL/rnatlp> |
|  |  | • RoBERTa [Robustly Optimized BERT Pretraining Approach] | LLM | free | <https://doi.org/10.48550/arXiv.1907.11692> |
|  |  | • RobotReviewer* | NLP, SupervisedML | free | <https://www.robotreviewer.net/> |
|  |  | • Robotics Process Automation [RPA] | NLP, ML | N/A | <https://doi.org/10.1109/ACCESS.2021.3072900> |
|  |  | • Rules_cochranereviews | NLP | free | <https://github.com/dsurian/rules_cochranereviews> |
|  |  | • Scholarcy* | N/A | $ | <https://www.scholarcy.com/> |
|  |  | • SLROnt Ontology | N/A | N/A | <https://doi.org/10.1007/s00607-023-01181-x> |
|  |  | • SENNA* ([Semantic Extraction using a Neural Network Architecture’] | NN | N/A | <https://doi.org/10.1371/journal.pone.0006393> |
|  |  | • Sparrow | LLM, ML | $ | <https://deepmind.google/> |
|  |  | • srBERT* [Bidirectional Encoder Representations from Transformers] | LLM, ML | free | <https://github.com/SEONCHOE/> |
|  |  | • SRDR+* [Systematic Review Data Repository] | N/A | N/A | <https://srdrplus.ahrq.gov/> |
|  |  | • Statistical package R* | N/A | free | <https://www.r-project.org/> |
|  |  | • SyRF* | NLP | free | [http://syrf.org.uk](http://syrf.org.uk/) |
|  |  | • Sysrev* | SupervisedML | $ | [https://sysrev.com](https://sysrev.com/) |
|  |  | • Switch | LLM | free | <https://huggingface.co/google/switch-c-2048> |
|  |  | • T5 | LLM | free | <https://huggingface.co/docs/transformers/en/model_doc/t5> |
|  |  | • Table Builder | N/A | N/A | <https://table-builder.com/> |
|  |  | • Text2Onto | DM, TM, NLP | N/A | <https://doi.org/10.1016/j.artmed.2006.07.012> |
|  |  | • Topictagger | N/A | N/A | <https://doi.org/10.1186/s12874-022-01805-4> |
|  |  | • Trialstat SRS* | N/A | $ | <https://trialstat.com/> |
|  |  | • ULMFit [Universal Language Model Fine-tuning] | LLM | free | <https://doi.org/10.12688/f1000research.51117.2> |
|  |  | • UniLM/UniLMv2 | LLM | free | <https://doi.org/10.12688/f1000research.51117.2> |
|  |  | • WebPlotDigitizer | N/A | N/A | <https://automeris.io/WebPlotDigitizer.html> |
|  |  | • word2vec | LLM, NLP | free | <https://doi.org/10.12688/f1000research.51117.2> |
|  |  | • Wordstat 8.0 | N/A | N/A | <https://gades-solutions.com/en/home/> |
| **Assess the quality of the studies** | **Critical appraisal** | • (Afzal et al., 2019) Framework | ML | free | <https://doi.org/10.2196/13430> |
|  |  | • AMSTAR Checklist* | N/A | free | <https://amstar.ca/Amstar_Checklist.php> |
|  |  | • ASSERT* [Automatic Summarisation for Systematic Reviews using Text Mining] Project Tools | TM | free | <https://www.nactem.ac.uk/assert/> |
|  |  | • Biomed-Summarizer | DL, NN | N/A | <https://doi.org/10.2196/19810> |
|  |  | • (Brassey et al., 2021) Tool | ML, NLP | free | <https://doi.org/10.1136/bmjebm-2018-111126> |
|  |  | • CONSORT-NLP* | N/A | free | <http://www.consort-nlp.org/> |
|  |  | • Covidence* | ML | $ | <https://www.covidence.org/> |
|  |  | • CrowdCARE* | N/A | free | <https://crowdcare.unimelb.edu.au/index.html?g=true&ts=1666711451071&page=> |
|  |  | • DALL-E* | LLM, NN | $ | <https://openai.com/research/dall-e> |
|  |  | • DistillerSR* | NLP, SupervisedML | $ | <https://www.distillersr.com/> |
|  |  | • EPPI-Reviewer* | NLP | $ | <https://eppi.ioe.ac.uk/eppireviewer-web> |
|  |  | • EvidenceGRADEr* | ML | free | <https://zenodo.org/records/5653587> |
|  |  | • ExaCT [EXtracting Accurate efficacy and safety information from ClinicalTrials.gov] | TM, ML | $ | <https://doi.org/10.1186/1472-6947-10-56> |
|  |  | • GRADEpro* | N/A | free | <https://www.gradepro.org/> |
|  |  | • GPT-4* | LLM | $ | <https://openai.com/research/gpt-4> |
|  |  | • JBI SUMARI* [System for the Unified Management of the Assessment and Review of Information] | N/A | $ | <https://sumari.jbi.global/> |
|  |  | • LaMDA* [Language Model for Dialogue Applications] | LLM | $ | <https://blog.google/technology/ai/lamda/> |
|  |  | • LLaMA* [Large Language Model Meta AI] | LLM | free | <https://llama.meta.com/> |
|  |  | • LBJ [Learning Based Java] | NLP, ML | N/A | <https://doi.org/10.1109/ICSC.2007.53> |
|  |  | • MetaPreg* | ML, NLP | N/A | <http://metapreg.org/> |
|  |  | • Multiclassifier Tool for Risk of Bias [MCRB] | ML | N/A | <https://doi.org/10.1109/CBMS49503.2020.00008> |
|  |  | • ORBIT Matrix Generator* | N/A | free | <https://www.outcome-reporting-bias.org/> |
|  |  | • PRISMA-DFLLM [Domain-specific Finetuned LLMs] | LLM | N/A | <https://doi.org/10.48550/arXiv.2306.14905> |
|  |  | • RAx [enagoRead]* | NLP, SupervisedML | $ | [https://raxter.io](https://raxter.io/) |
|  |  | • RobotReviewer* | NLP, SupervisedML | free | <https://www.robotreviewer.net/> |
|  |  | • Robvis | N/A | free | <https://www.riskofbias.info/welcome/robvis-visualization-tool> |
|  |  | • (Ruiz et al., 2021) MTTR [Method and Tool for Generating Table of Relevance] | TM | free | <https://repository.londonmet.ac.uk/id/eprint/6931> |
|  |  | • (Sarker et al., 2015) Unnamed Framework | SupervisedML, NLP | N/A | <http://dx.doi.org/10.1016/j.artmed.2015.04.001> |
|  |  | • Segment Anything Model* [SAM] | LLM | $ | <https://segment-anything.com/> |
|  |  | • Sentiment Analysis | N/A | N/A | <https://doi.org/10.1016/j.jbi.2023.104389> |
|  |  | • Sysrev* | SupervisedML | $ | [https://sysrev.com](https://sysrev.com/) |
|  |  | • Systematic Review Assistant | N/A | N/A | <https://github.com/IEBH/SRA> |
|  |  | • Vision Transformer | LLM | N/A | <https://doi.org/10.7759/cureus.43023> |
| **Combine the data** | **Meta-analysis** | • (Brassey et al., 2021) Tool | ML, NLP | free | <https://doi.org/10.1136/bmjebm-2018-111126> |
|  |  | • Comprehensive Meta‐Analysis (CMA) | N/A | $ | <https://meta-analysis.com/> |
|  |  | • MAGICapp* [Evidence Ecosystem Foundation] | N/A | N/A | <https://magicevidence.org/> |
|  |  | • [Open]Meta-analyst* | N/A | free | <http://www.cebm.brown.edu/openmeta/> |
|  |  | • Meta-DiSc* | N/A | free | <http://www.metadisc.es/> |
|  |  | • MetaPreg* | ML, NLP | N/A | <http://metapreg.org/> |
|  |  | • MetaWin* (free, ) | N/A | free | <https://www.metawinsoft.com/> |
|  |  | • MetaXL* | N/A | N/A | <https://www.epigear.com/index_files/metaxl.html> |
|  |  | • NetMetaXL* | N/A | free | <https://www.netmetaxl.com/> |
|  |  | • RevMan* | N/A | $ | <https://training.cochrane.org/online-learning/core-software/revman> |
|  | **Figures visualisation** | • ALBATROSS | N/A | N/A | <https://doi.org/10.1002/jrsm.1239> |
|  |  | • BUGS/OpenBUGS/WinBUGS [Bayesian inference Using Gibbs Sampling] | N/A | N/A | <https://www.mrc-bsu.cam.ac.uk/software/bugs/> |
|  |  | • (Brassey et al., 2021) Tool | ML, NLP | free | <https://doi.org/10.1136/bmjebm-2018-111126> |
|  |  | • DistillerSR* | NLP, SupervisedML | $ | <https://www.distillersr.com/> |
|  |  | • EPPI-Reviewer* [EPPI-Mapper] | NLP | $ | <https://eppi.ioe.ac.uk/eppireviewer-web> |
|  |  | • Gephi* | N/A | free | <https://gephi.org/> |
|  |  | • JAGS [Just Another Gibbs Sampler] | N/A | N/A | <https://mcmc-jags.sourceforge.io/> |
|  |  | • NestedKnowledge* | ML | free | <https://nested-knowledge.com/> |
|  |  | • PRISMA 2020* | N/A | N/A | <http://www.prisma-statement.org/?AspxAutoDetectCookieSupport=1> |
|  |  | • PRISMA Flow Diagram Generator | N/A | free | <https://estech.shinyapps.io/prisma_flowdiagram/> |
|  |  | • Prisma diagram R* | N/A | free | <https://github.com/prisma-flowdiagram/PRISMA2020> |
|  |  | • PRISMAstatement* | N/A | N/A | <http://www.prisma-statement.org/PRISMAStatement/> |
|  |  | • Revtools [R package]* | TM, NLP | free | <https://cran.rproject.org/package=revtools> |
|  |  | • ROSES* [RepOrting standards for Systematic Evidence Syntheses] flowchart | N/A | N/A | <https://www.roses-reporting.com/flow-diagram> |
|  |  | • SWIFT Active Screener* | ML | $ | <https://www.sciome.com/swift-activescreener/> |
|  |  | • yEd Graph Editor* | N/A | $ | <https://www.yworks.com/products/yed> |
|  |  | • WebPlotDigitizer | N/A | N/A | <https://automeris.io/WebPlotDigitizer.html> |
| **Discuss and conclude overall findings** | **Summary of Findings/Report** | • Alpaca | LLM | N/A | [https://doi.org/10.1093/bib/bbad493 https://doi.org/10.21203/rs.3.rs-3483777/v1](https://doi.org/10.1093/bib/bbad493) |
|  |  | • BERT [Bidirectional Encoder Representations from Transformers] (LLM, free,) | LLM | free | <https://research.google/pubs/bert-pre-training-of-deep-bidirectional-transformers-for-language-understanding/> |
|  |  | • BioBERT | TM, LLM | N/A | <https://hpc.nih.gov/apps/BioBERT.html#:~:text=BioBERT%20is%20a%20biomedical%20language,extraction%2C%20question%20answering%2C%20etc.> |
|  |  | • BioMedLM* | LLM | N/A | <https://crfm.stanford.edu/2022/12/15/biomedlm.html> |
|  |  | • (Denzler et al., 2021) Artifact | ML, TextAnalytics | N/A | <https://aisel.aisnet.org/amcis2021/art_intel_sem_tech_intelligent_systems/art_intel_sem_tech_intelligent_systems/4> |
|  |  | • ChatGPT* [based on GPT-3.5] | LLM | free/$ | <https://openai.com/chatgpt> |
|  |  | • ClinicalBERT | LLM (BERT) | N/A | <https://doi.org/10.48550/arXiv.1904.05342> |
|  |  | • Clinical Camel | LLM | free | <https://doi.org/10.48550/arXiv.2305.12031> |
|  |  | • DistillerSR* | NLP, SupervisedML | $ | <https://www.distillersr.com/> |
|  |  | • FLAN-T5 | LLM | N/A | <https://huggingface.co/docs/transformers/en/model_doc/flan-t5> |
|  |  | • FLAN-UL2 | LLM | N/A | <https://huggingface.co/docs/transformers/en/model_doc/flan-ul2> |
|  |  | • Galactica | LLM | free | <https://gpt3demo.com/apps/meta-galactica-ai> |
|  |  | • GatorTron* | LLM | free | <https://catalog.ngc.nvidia.com/orgs/nvidia/teams/clara/models/gatortron_og> |
|  |  | • ChatDoctor* | LLM | $ | <https://www.chatdoctor.com/> |
|  |  | • (Chat)GPT-3* | LLM | $ | <https://openai.com/blog/gpt-3-apps> |
|  |  | • InstructGPT | LLM | $ | <https://openai.com/research/instruction-following> |
|  |  | • JBI SUMARI* [System for the Unified Management of the Assessment and Review of Information] | N/A | $ | <https://sumari.jbi.global/> |
|  |  | • LLaMA-2* [Large Language Model Meta AI] | LLM | N/A | <https://llama.meta.com/llama2/> |
|  |  | • LiSA* [Literature Search Application] | DL, NER, LLM (BERT) | N/A | <https://doi.org/10.1186/s12911-022-02085-0> |
|  |  | • LongT5 model | LLM | N/A | <https://huggingface.co/docs/transformers/en/model_doc/longt5> |
|  |  | • MAGICapp* [Evidence Ecosystem Foundation] | N/A | N/A | <https://magicevidence.org/> |
|  |  | • MedAlpaca | LLM | N/A | <https://github.com/kbressem/medAlpaca> |
|  |  | • Med-PaLM 2* | LLM | $ | <https://sites.research.google/med-palm/> |
|  |  | • MetaMap | ML | N/A | [https://doi.org/10.1016/j.jbi.2010.04.001 https://doi.org/10.1109/APSEC.2017.10 https://doi.org/10.1186/s13643-024-02592-3](https://doi.org/10.1109/APSEC.2017.10) |
|  |  | • NestedKnowledge* | ML | free | <https://nested-knowledge.com/> |
|  |  | • PaLM* | LLM | $ | <https://ai.google/discover/palm2/> |
|  |  | • PERSIVAL [PErsonalized Retrieval and Summarization of Images, Video and Language] | N/A | N/A | <https://doi.org/10.1016/j.artmed.2004.07.018> |
|  |  | • PMC-LLaMA | LLM | N/A | <https://doi.org/10.1093/jamia/ocae045> |
|  |  | • PubMedBERT | N/A | N/A | [https://doi.org/10.17632/ccfnn3jb2x.1 https://doi.org/10.1038/s41591-023-02448-8](https://doi.org/10.17632/ccfnn3jb2x.1) |
|  |  | • ReVis | VisualTM | N/A | <https://doi.org/10.1007/s00607-023-01181-x> |
|  |  | • RevMan [Review Manager] HAL* | N/A | N/A | <https://schizophrenia.cochrane.org/revman-hal-v4> |
|  |  | • SRA Replicant Writer* | N/A | free | <https://sr-accelerator.com/#/replicant> |
|  |  | • Text2Onto | DM, TM, NLP | N/A | <https://doi.org/10.1016/j.artmed.2006.07.012> |
|  |  | • Turing-NLG* [Turing Natural Language Generation or CoPilot] | LLM | $ | <https://turing.microsoft.com/> |
|  |  | • Vicuna (LLM) | LLM | N/A | <https://doi.org/10.21203/rs.3.rs-3483777/v1> |
| **Systematic review (update)** | | • Trial2rev | SemiSupervisedML | free | <https://github.com/evidence-surveillance/trial2rev> |
| **Dissemination** | **Publication** | • BART | LLM | free | <https://huggingface.co/docs/transformers/en/model_doc/bart> |
|  |  | • BioGPT | LLM | N/A | <https://github.com/microsoft/BioGPT> |
|  |  | • ChatGPT* [based on GPT-3.5] | LLM | free/$ | <https://openai.com/chatgpt> |
|  |  | • Manuscript Matcher* | N/A | $ | <https://endnote.com/manuscript-matcher/> |
|  |  | • SciFive | LLM | N/A | <https://doi.org/10.48550/arXiv.2106.03598> |
|  |  | • T5 | LLM | N/A | <https://huggingface.co/docs/transformers/en/model_doc/t5> |
|  | **SLR database** | • BEME [Best Evidence Medical Education] Collaboration | N/A | N/A | <http://www.bemecollaboration.org/> |
|  |  | • Campbell Collaboration* | N/A | N/A | <https://www.campbellcollaboration.org/> |
|  |  | • Cochrane Library* | N/A | N/A | <https://www.cochranelibrary.com/> |
|  |  | • Epistemonikos* | N/A | N/A | <https://www.epistemonikos.org/> |
|  |  | • JBI EBP* | N/A | N/A | <https://jbi.global/jbi-ebp-database> |
|  |  | • MAGICapp* [Evidence Ecosystem Foundation] | N/A | N/A | <https://magicevidence.org/> |
|  |  | • PEDro* [Physiotherapy Evidence Database] | N/A | N/A | <https://pedro.org.au/> |

Updated from Jimenez and colleagues infographic [5] to outline data science methods that are either open-source/ free for access (free) or proprietary ($), requiring payment for access, as well as whether they are being only developed or also implemented (*). We provide a hyperlink to access further information regarding each tool and platform – all according to information found in the 137 included studies and reference lists. (NB. those without a link have been identified in one of the ES included whose reference is listed in the **Supplementary Table S3**).

### **Supplementary Findings**

**Regional Specialisation – Findings per Region and Country.**

**I – Asia-Pacific**

We identified that Australian authors have been either developing and/or implementing particularly TM, ML and NLP data science methodological approaches, together and/or separately, to automatise all ES steps with eight case studies/reports [6–13], two non-SLRs on ML models [14, 15] and three text/opinion publications on various AI approaches [16–18].

Chinese authors have systematised a variety of data science methods in one very recent SLR [19], and is deploying DM in two case-studies/reports [20, 21] to optimise search, extraction and meta-analysis ES steps. While one Indian group of researchers [22] have recently presented their case-study to explore DistillerSR – an ML and NLP tool – to automatise the screening step of ES, and another described a comprehensive SLR synthesising especially ML and NLP approaches for data extraction automation [23].

Japanese authors have been specialising in LLMs, either alone [24] or in recently-established partnership with UK researchers [25] to automatise screening. While Malaysian authors have been systematising evidence on TM tools and techniques to optimise all ES steps under a narrative review [26], or partnering with Middle-Eastern researchers to deploy LLMs (i.e. ChatGPT) for a very recent narrative review on LLMs approaches to automatise all ES steps [27].

New Zealand authors have developed two case-studies to describe the 2023 new PRISMA tool for LLMs’ report [28] and a ML technique to automatise the study selection step [29]. One group of Korean authors have reported two case studies on the development of a ML approach to automatise study search, screening, critical quality appraisal and summary steps [30] and a deep NN model to optimise study protocol, searching, screening, critical quality appraisal and synthesis [31], and another group have reported on the implementation of srBERT – a ML model to optimise study screening [32]. Finally, Singaporean authors partnered with UK and USA researchers to report on a non-SLR on LLMs being both recently developed and/or implemented to automatise summary step of ES [33].

**II – Europe**

In Austria, we identified a case study reporting on a TM approach under development to semi-automatise the extraction step from ‘living’ guideline development [34], and an analytical cross-sectional study that already implements a NN model to automatise screening step of ES [35]. French authors reported a case-control study on a very recent ML model that they are implementing to automatise all meta-analysis steps [36], and yet another recent case study on a DL tool – i.e., LiSA (Literature Search Application) – that they are implementing to optimise study search, extraction and synthesis steps of ES [37]. Another French group systematised very recently published evidence on both ML and NLP tools and techniques that are being both developed and/or implemented to automatise study search, deduplication, screening and extraction steps of ES in a mapping review [5].

We identified three case study from German authors reporting on a very recent DM tool (KIETA) [38] that is being developed and the implementation of a TM tool (Adobe Acrobat Pro DC) [39] and a NN tool (SENNA – Semantic Extraction using a Neural Network Architecture) [40] to automatise data extraction step of ES. Other three case studies reporting the implementation of a NLP tool (XplorMed) [41] to optimise study search step, a TM tool (PubFinder) [42] automatising screening step, and the development of a recent ML Artifact [43] optimising search, reference management, screening, visualisation and synthesis steps of ES. We have also identified two very recent SLRs summarising ML approaches [44] and LLMs [45] being implemented to automatise search, screening and selection, and study protocol, synthesis and publication steps of ES, respectively.

In Greece, we have identified a group of authors, who have recently partnered with UK and USA researchers, reporting a case study on a ML, DL and NLP tool (Pythia) they are developing to optimise study search and screening steps of ES [46].

In the Netherlands, researchers have reported one non-SLR systematising TM approaches optimising mainly the search step of ES [47]. Dutch authors have also reported on two case studies of ML approaches being developed to optimise the screening step of ES [48, 49]. Other Dutch researchers have partnered with Spanish co-authors to develop a DM and TM approach to automatise extraction, summary/synthesis steps of ES [50], and with UK and USA co-authors to implement a ML tool (RobotReviewer) that optimises the critical quality appraisal step of ES [51].

A Norwegian group of authors have also reported on the implementation experience of RobotReviewer, under a randomised-controlled trial [52] to automatise the critical quality appraisal step of ES. Whereas, a Norwegian case study reported on a DL tool (NIPH coding workflow) that is being developed to optimise the screening step of ES [53].

Spanish researchers have reported on recent three case studies both developing and implementing ML approaches to automatise study planning, protocol, search, screening, selection, extraction, critical quality appraisal, synthesis and dissemination, alone [54] and in partnerships with Portuguese [55] and UK coauthors [56].

Swedish authors have also reported a very recent comparative study on a series of ML, TM and NLP tools and approaches that are being implemented to automatise study search, screening, extraction and synthesis, alone [57], and another very recent case study on a DL tool (RoBERTa) that is being developed, in partnership with UK and USA coauthors [58], to optimise study search, screening and selection steps of ES.

Swiss authors have reported a very recent case study on a ML approach to optimise screening step of ES [59], another very recent qualitative study on a ML and NLP approach that is being implemented to automatise study searching, screening and selection steps [60], and yet other very recent abstracts reporting various commercially available AI tools and platforms (EPPI-Reviewer, DistillerSR, AbstrackR, Rayyan, Python packages) that are being implemented to optimise screening [61] and extraction steps of ES [62].

In the UK, authors have reported eleven case studies: one on a DM approach that is being developed to automatise study protocol step [63]; three distinct TM tools – one that is being implemented to automatise screening step [64], ASSERT project that is being implemented to optimise study search, screening and synthesis steps [65], and Canonical Model that is being developed to automatise data extraction step of ES [66]; four ML approaches – Lingo3 being implemented to automatise study searching [67], and other three being developed to optimise reference management [68] and screening [69, 70]; one more recent ML and NLP approach to automatise study searching, critical quality appraisal, meta-analysis and visualisation [71]; NNs tools (QA-BERT, SCIBERT, BERT) that are being developed to automatise study protocol [72]; and a very recent LLM tool (Bio-SIEVE) that is being both developed and implemented to optimise screening step of ES [73]. UK authors have also recently systematised quite a few evidence into: a Living SLR [72] on ML, DL, NNs, NLP and LLMs being developed and implemented to automatise extraction step; a mapping review [74] on a TM tool (VOS Viewer) being implemented to optimise study protocol and search steps; two narrative reviews reporting on a large series of ML approaches being implemented to automatise study search, screening (Marshall et al., 2020 Semi-automated), extraction and critical quality appraisal [75]; a scoping review on various AI tools that are being implemented to optimise screening step [76]; two opinion pieces reporting on a TM and ML tool (EPPI-Reviewer) [77] and a Systematic Online Living Evidence Summaries (SOLES) [78] that are being implemented to automatise all ES steps; a conference abstract reporting a ML software that is being developed to optimise the screening step [79]; and a grey literature evaluation study of LLMs (T5, SciFive,BART, BioGPT, ChatGPT) to automatise the publication step of ES [80].

**III – Middle-East**

Qatar authors have published a very recent editorial on a series of LLMs that are being implemented to automatise the ES critical quality appraisal step [81]. Another Qatar group has also partnered with Bahrain researchers to report a case study on a ML tool (Rayyan) that is being both developed and implemented to optimise screening step [82]. Whereas a Saudi Arabian and USA group of researchers have partnered to report on a very recent non-systematic overview of LLMs, including ChatGPT, that are being both developed and implemented to automatise extraction and synthesis steps of ES [83].

**IV – North America**

In Canada, authors have reported six case studies on: a language processing system (Best-evidence Retrieval and Delivery, BiRD) system that was being developed to automatise study search step [84]; a TM tool (ExaCT) that was being developed to optimise extraction step [85]; a ML factorised version of the complement naïve Bayes (FCNB) implemented that was being developed to automatise screening step [86]; a parallel NLP classifier that was being developed to optimise screening step [87, 88] in partnership with USA coauthors; a very recent NLP strategy (Ananse) that had been implemented to automatise study search step [89]; and a very recent LLM (OpenAI GPT DC) that is being implemented to optimise study screening step pf ES [90]. Two other groups of Canadian authors reported a series of five evaluation studies on ML tools that have been implemented to automatise study screening (AbstrackR, DistillerSR and RobotAnalyst) [91–94] and critical quality appraisal (RobotReviewer) [95]). Another group of Canadian authors [96] recently published a qualitative study outlining a series of AI tools that have been implemented to optimise study protocol and search (TheoryOn, Litbaskets, LitSonar), screening (ASReview and ADIT), critical quality appraisal (statistical software packages and RobotReviewer), extraction (WebPlotDigitizer and Graph2Data) and synthesis (RevMan).

USA authors have reported a total of twenty-two case studies on: two DM approaches that had been developed to optimise study search (Search Builder 1.0) [97] and screening [98] steps; a series of TM tools that had been implemented to automatise study search step – Reflective Random Indexing [99], LitSearchR [100], Anne O'Tate , BiblioShiny, Carrot2, CitNetExplorer, EndNote, Keyword‐Analyzer (formerly TextAlyser), Lingo3G (Carrot), Lingo4G, MeSHonDemand, PubReMiner, TerMine, Text Analyzer, Tm for R, VosViewer, Voyant, and Yale MeSH Analyse [100] – and a TM and ML tool (SWIFT-Reviewer) that had been implemented to optimise study screening step; a series of ML tools and approaches that had been both developed and implemented to automatise study search step – LitSuggest [101], unnamed approach [102] – screening – AbstrackR [103, 104], RapidMiner v.52 [105], DistillerAI [106], RobotAnalyst [104], RCT Tagger [107], and unnamed approach [102] – selection – LIbSVM classifier [108, 109], and unnamed approach [102] – and recently, extraction – DextR [110]; a NLP tool (Computational-Linguistic Approaches to Indexing and Retrieval of Text, CLARIT) that had been implemented to automatise study screening step [111]; a series of LLM tools that are being both developed and implemented to optimise summary/ synthesis step – Long T5 model [112] and ChatGPT 3.5 [113], which also automatises study search, extraction and synthesis steps of ES [113]; PDFBox Tool that is being implemented to optimise extraction step [114], as well as three other tools [115] – ReviewManagement software (RevMan), Trialstat SRS and EPPI (The Evidence for Policy and Practice Information and Coordinating] Centre) Reviewer.

US researchers have also reported on their experiences with a ML tool (MiSearch) that had been developed to automatise study search step [116], and several AI tools [117] that had been implemented to optimise study screening – DistillerSR, EPPI-Reviewer and AbstrackR – extraction – Systematic Review Data Repository (SRDR) and DistillerSR – and meta-analysis – Comprehensive Meta-Analysis, Meta-Analyst, and Gibbs Sampling (BUGS) implementations, such as WinBugs/OpenBugs or Just Another Gibbs Sampler (JAGS). Two groups of USA specialists have also reported an experimental study on an ML algorithm that had been implemented to automatise study screening and selection steps of ES [118], and a support guide [119] to help developers and users choose amongst various AI tools that are being implemented to optimise all ES steps (Cadima, Covidence, Colandr, DistillerSR, EPPI-Reviewer Web, Giotto Compliance, JBI SUMARI, LitStream, Nested Knowledge, PICOPortal, Revman Web, SRDB.PRO, SRDR+, SyRF and SysRev), study screening (AbstrackR, Rayyan, RobotAnalyst, SWIFT-Active Screener and SR Accelerator), extraction (Data Abstraction Assistant, RobotReviewer and SRDR), and figure visualisation (COVID-NMA).

Finally, we also identified US-based grey literature publications reporting on a series of LLM tools that are currently being implemented to automatise summary/ synthesis step of ES – AI Writer, BERT (Bidirectional Encoder Representations from Transformers) by Google), GPT-3 Generative Pre-trained Transformer 3, by OpenAI), and Turing-NLG (Turing Natural Language Generation) by Microsoft [120]; ChatGPT 3.5 [121]; FLAN-T5 ,FLAN-UL2, Alpaca, Med-Alpaca, Vicuna, Llama-2, GPT-3.5 and GPT-4 [122].

**V – South America**

Brazilian specialists have reported one case study on a TM approach (visual text mining, VTM) that had been developed to automatise the ES study selection step [123]. Other three groups of authors in Brazil have also very recently systematised evidence in: a SLR [124] on several ML tools that had been implemented to optimise study screening step of ES – AbstrackR (TM), EPPI Reviewer, DistillerSR, RobotAnalyst, DistillerAI, Twister (TM), Swift Review, AFLEX Tag, Spark-Text (TM), and GATE (<http://gate.ac.uk>) – as well as two non-SLRs with a large series of TM and ML tools [125], and LLM platforms (Elicit ([www.elicit.org](http://www.elicit.org)), SciSpace Copilot (<https://typeset.io/>), ResearchRabbit (<https://www.researchrabbit.ai/>) and ChatGPT (<https://openai.com/chatgpt>) [126] that are currently being developed and implemented to automatise all ES steps.

Finally, in Chile, a group of authors has very recently published an editorial to discuss the roles and challenges of ChatGPT as a LLM that is currently being implemented to automatise all ES steps [127].

### **Supplementary Discussion.**

**Barriers and Facilitators to AI Tools and Platforms Development and Implementation**

Development and implementation specialists have reported a series of concerns regarding barriers for when deploying AI tools and platforms to optimise **all** ES steps.

**I – ES Planning and Protocol Writing**

First, when it comes to creating new strategies to face barriers for automatising **ES planning** step, **'citizen science'** resources and platforms provide an opportunity of crowdsourcing part of a Living ES workload, and piecing Living SLRs processes into smaller and clearly-defined ‘microtasks’, with the aim of increasing overall speed and efficiency gains [128].

Second, regarding **protocol writing**, specialists have reported a further barrier to a fully automated approach due to inaccurate detection of PICO elements in the automatically annotated (i.e., single labelling) PubMed training data. Therefore, a publication may describe several interventions in different populations, testing different comparators and measuring those populations on different outcomes, but it remains difficult to determine how many matches indicate that a PICO element is part of a study described within a publication, rather than a match to a reference to other research, for example [78, 129]. On the other hand, there are a few AI tools such as SWIFT-Review [130], BioIngine [63] and BERT models [129] that have been developed and implemented with human tagging and annotation at certain points, which should provide better performance if not developed as a stand-alone organisational interface. However, these are still to be further tested.

**II – Search**

Third, developers and implementers detailed concerns regarding barriers for when deploying AI tools and platforms to optimise ES **study search** have focused on outlining that all AI tools and platforms currently available still require more testing to fine-tune a series of issues, suggesting that final approval of the evidence by a human is crucial to avoid interpretations if wrong decisions are made by the system [30], particularly for **de-duplication** and **management of records** [128], even with promising new tools such as Litsearchr [100]. This is mainly because: a) tools such as LitSuggest can currently only process indexed publications , therefore, non-peer reviewed publications that do not have abstracts cannot be processed and accuracy is lost [101]; b) leveraging ontologies, such as the Unified Medical Language System (UMLS), and knowledge databases (electronic resources that contain curated information from data repositories) to better enrich the questions used in the search queries [46].

Fourth, much of the AI tools only do a single ES step automation, hence the need for human manual input to shift output from one tool to another, as well as the need for model flexibility and robustness to handle large data sets [16] and provide the adequate interoperability [65], which we are still to observe even for more comprehensive AI models, such as ChatGPT [131], RoBERTa [58] – whose performance against retrospective data remains insufficient and can introduce selection bias, as well as not perform uniformly across products – Sherlock (limited search engine for trial registries at clinicaltrials.gov), Quick Clinical (limited federated meta-search engine that is not optimised for SLRs) [14, 15] and LiSA [37].

Fifth, there is the issue of those tools and platforms whose efficacy may depend on the user type, hence, the need for expert users to leverage a tool’s full functionality [43] and produce more tailored interfaces to the needs of information specialists and decision-makers [132]. Fifth, given distinct resources and expertise distribution across HICs and LMICs, there is also the concentration of publications and AI tools and platforms development and implementation across only five countries (USA, UK, Australia, Canada and China), and the consequent absence of publications from African and many Latin American countries, likely due to poorer research support and lower prioritisation of policies that address the more basic needs of the countries. Hence, the dramatical general differences between HICs and LMICs with regards to AI tools and platforms development and implementation glocally [125].

Sixth, following such key multicultural shortcomings, several tools – such as BIBOT, RobotReviewer and Cochrane RCT Classifier – have only been developed for studies published in the English Language and cannot be generalised for other languages [14]. An essential reason why interoperability amongst distinct AI tools and platforms is key, especially for translation – an option widely covered by Google Translate [14, 85]. Nevertheless, algorithm transparency is needed and specialists have been arguing for black-box models to remain in the past [54, 128]. In this sense, a manually created coding tool developed from a coherent conceptual framework that can clearly demonstrate where there are research gaps should become the norm [67]. Therefore, increasingly widespread use of DL algorithms and architectures has been accelerated by massive investments made by large tech companies, along with the compilation of large sets of data (big data), the development of open-access software libraries and frameworks, and the accessibility of computing power [125].

Seventh, the creation of tool connecting pipelines will allow for greater automation across multiple tasks [8], especially enabling manual features that are compatible with Living ES workflows, whilst still supporting multi-user collaborative web-environments, such as Covidence, Abstrackr [128] and ‘Cochrane Crowd data’ [11] – all widely used across HICs and LMICs.

**III – Screening and Selection**

Eighth, despite such new more democratic tools and platforms, there still remains **access barriers** as certain tools – such as LiteRev [60] and SWIFT-Review [130] for **screening** and **selection** steps – are currently **limited to open-access databases** that provide free application programming interface (APIs) to abstract or full-text papers since **subscription databases** such as Embase or Web of Science do not provide API access, or do not allow for TM and ML analysis [60].

Additional development and implementation barriers regarding the ES **screening** and **selection** steps, as reported by specialists, mainly ranged around specific (especially ML-based) [70, 78] algorithms, tools and platforms limitations: a) within the (Bidirectional Encoder Representations from) Transformer (BERT) family of models, such as srBERT, performance depended on the size and class ratio of the training dataset, therefore, securing datasets of sufficient quality is essential, which may pose challenges to both developers and implementers [5, 11, 32]. This is a barrier also for **data extraction** [75] ES step; b) when it comes to updating a standard ES or evolving it into a ‘living’ one, much can change, including the team, the searches, and even aspects of the research question, which further complicates **screening**, **selection** and **extraction** automation [105]; c) **screening** and **selection** have been recognised as the most time-consuming steps in ES – as such, **screening** and **selection** **workload** is a widely known **barrier** that remains to be widely assessed with larger multi-stakeholders samples as well as for SLRs and Living ES processes beyond the health field before specialists can assert whether time and cost savings introduced by tools such as Research Screener may generalise well to other disciplines [7, 54, 69]; d) following this issue of underprovision of ‘living’ or updatable outputs, **screening** has been identified as the sole ES step being automatised (54% of tools) [119].

Therefore, developers and implementers outline that certain performance outcomes (such as feature count) does not equate to value or usability, failing to capture benefits of simple platforms, such as ease of use, effective user interface, alignment with established workflows [124], or relative costs [119].

**IV – Reference Management and De-duplication**

Ninth, specialists were disappointed with overall performance of several tools – i.e. DistillerAI [106], Abstrackr, RobotAnalyst, DistillerSR [92, 93] and PubFinder [42] – for consequential false-positive decisions (i.e., excluding relevant records), which could affect the validity of a systematic review, requiring not only human manual input [93] – especially **reference management** and **de-duplication** [94] – but also higher computing time [42]. Nevertheless, some authors identified a relatively minimal percentage of human-machine conflicts for these same tools, implying that a machine could be employed as a second reviewer when conducting SLRs, hence, reducing time to complete, workload and enabling swifter decision-making [61, 62].

Regarding costs, the tenth barrier comprises widespread uptake of priority-ranking methods – such as SWIFT-Review [130] or RapidMiner [105] – and how investment in future versions of such computer-assisted decision support system based on SLR teams and solo reviewers could substantially reduce screening and selection burden, while delivering quality assurance both by confirming concordant decisions and naming studies associated with discordant decisions for further human validation.

In this sense, although investments in automatising screening with OpenAI’s Generative Pre-Trained Transformer (ChatGPT) version 3.5 greatly outperformed previous ML-based methods and showed sensitivity close to human screening performance [24], maintaining human input in the loop still remains not only advantageous but quite fundamental [12]. As such, since humans and machines have different capabilities, developers can reconceive both ES individual steps as well as their ordering when machines undertake them [8] to understand at which point it is ‘safe’ for the human reviewer to stop manual screening. For now, AI platforms (such as AbstrackR, RobotAnalyst, and EPPI Reviewer) could safely be used as a second screener to prioritise abstracts for manual selection [75], as less experienced reviewers acceptability was generally more positive, as they used RobotReviewer more flexibly. Nevertheless, all prioritised human input and human-to-human interaction to even semi-automatised ES steps [52].

Hence, on the eleventh barrier, developers have also discovered some recent works exploring new ML approaches – arguably one of the best techniques at present for ES automation [5] – in which humans can be more involved (such as FASTREAD and FAST2), because strongly relying on publication content implies that the classifier can only use lexicon of one field to make decisions, missing those publications adopting different or emerging terminology, or simply those covering new topics – i.e. by analysing related research communities, including co-authorships and cross-references, all necessary to identify emergent topics for which a standardised terminology has not been comprehensively developed yet [54]. Another reason why the twelfth barrier lies in technical issues from matching dataset with the experimental setup of the original publications, and executing the original source code, to understanding that code availability alone does not guarantee a reproducibility [35, 48] in a wide variety of (sub)fields [12], even within the health area [133]. As such, the thirteenth barrier comprises the gap in high-performing classifiers to identify field-specific studies, for example, in diagnostic test accuracy studies because training data are not available [88].

Overcoming scepticism [134] and specific ethical concerns [127] towards automation involves tackling the fourteenth barrier, which is improving technical evaluations parameters, especially sensitivity (recall), not only in widely implemented ML tools and platforms – such as AbstrackR, RobotAnalyst [104, 135], DistillerSR [22], RCT Tagger, RobotReviewer, RobotSearch, SWIFT-Active Screener, SWIFT-Review and SRA-Helper [107] but also LLMs – such as ChaGPT [73] to avoid hallucinations by poor zero-shot performance, and reduce human workload and time, as opposed to promising new findings by including few-shot prompting for Bio-SIEVE [73]. Therefore, the fifteenth barrier is that we need new scholarship outlining acceptable error rates when applying this wide range of data science methods assemblage in both open-source and commercial tools and platforms to optimise decision-support technology in real-life workflows for screening and selection ES steps [53].

The sixteenth barrier outlines that we also need data quality (in terms of volume [48] and validation) parameters to be improved and implemented [25], because, according to developers, decisions made by these AI tools and platforms still need to be combined with those of a reviewer to minimise errors, therefore, semi-automation is recommended meanwhile [49, 124]. The seventeenth barrier points to provision of clear standards and guidelines should be globally deployed in capacity building initiatives with multi-professional teams, as supported by PAHO [136], WHO [137, 138] and academic experts from multidisciplinary fields [27, 33, 125, 139–144]. In this direction, Revtools R packages constitutes an interesting initiative comprising several developers who are working together to construct an integrated package to guide users through a standard SLR workflow, while also focusing on building new software to integrate existing tools, by filling in the gaps between them for screening, selection and visualisation of textual data sources [13].

**V – Data Extraction**

Alongside summary/ synthesis, **data extraction** is the ES step where more LLMs have been developed and implemented. The eighteenth barrier to development and implementation of AI tools to optimise ES steps that we identified was that ChatGPT can effectively generate research questions and suggest search terms but cannot appropriately extract full-text data without human guidance autonomously [83] due to: dependence on input data, limited access to real-time data, length of prompts; token and memory limitations [131]. Then, Baviskar and colleagues [23] identified a series of barriers, namely: nineteenth barrier – *data* related – noisy poor quality, data diversity, variation in text data (especially unstructured data, as extraction adds incorrect text data in the form of noisy data, and those generated from multiple sources is non-standard and has different formats (i.e. diversity), which can also be a major issue for traditional extraction techniques, as well as lack of enough data and poor-quality data); twentieth barrier – *entities* related – ambiguities in entities, semantic and contextual relationships among entities, domain specific entities (extracting information from a highly ambiguous language such as Arabic, especially without creating a dictionary, is challenging, because semantics and contextual relationship among named entities remain challenging for current extraction techniques; domain specific entities poses another challenge as, for example, specific biomedical datasets domain entities differ from any other domain dataset); twenty-first barrier – *language* related – different languages, poor morphology (selection of the appropriate named entity recognition (NER) technique [38, 50]; lack of a large labelled corpus is another challenge; creating huge and manually labelled data is time-consuming and tedious task); twenty-second barrier – *type of unstructured documents* related – various layout, table data, multi-page structure (unstructured documents are of a different type, form, and layout by nature and extraction techniques should classify these documents by their type and nature; process and enhance the quality of the scanned documents to solve low-quality scanner or mobile devices; multi-page unstructured documents consisting of tables with data spanning across different pages also complicate retrieval of correct target data from document); twenty-third barrier – *existing datasets* related – poor quality (skewed, noisy; old datasets; blur, variations in lightning conditions), domain specific (task specific; handwritten character dataset; printed character dataset; language specific dataset; absence of details like labelling, annotations), data validation/quality assessment techniques (Cohen's Kappa; Chi Square Test; K-Fold Cross Validation), and privacy issues (confidentiality issues/ dataset owned by private companies/ government; very limited publicly available datasets).

The twenty-fourth type of barrier relates to usability, as certain extraction tools’ interface were found to be cumbersome by implementers – such as Review Manager (RevMan) – even though it facilitates ES writing and reference management in a uniform framework, further providing statistical analysis [115]. Nevertheless, although extraction remains a complex cognitive task, even partial automation may reduce the expertise required to complete this task, further reducing errors, saving time [15] and significantly reducing human intervention in time [23]. As such, key strengths across the field include collaborative mechanisms (offered by 83% tools) and easy, free access (71%), as outlined by the top four tools in terms of number of features offered – i.e., Giotto Compliance, DistillerSR, Nested Knowledge, and EPPI-Reviewer [119].

According to a recent expert SLR, such remarkable growth in publications over the last five years has been due mainly to **advances in DL approaches**, which have become increasingly more accurate and efficient in various scientific domains but particularly in unstructured biomedical data interpretation such as text and imaging [125]. Therefore, despite the trend to more powerful AI tools and platforms in practice, their implementation scope remains limited by a number of factors, but mainly their focus on ES screening and selection steps and the requirement of human intervention due to lack of system sensitivity [125]. In this sense, innovative tools such as KIETA [38] are offering interesting solutions especially regarding scientific domain agnosticism up until the ES data extraction step, which is based upon richer ontology [50]. A final twenty-fifth barrier in **data extraction** relates to **all other ES steps being automatised by powerful LLMs**: their likelihood to produce toxic, biased, or harmful content for humans, since the large corpora used for model training could contain both high-quality and low-quality data. It is therefore essential to align LLMs to generate helpful, honest, and harmless for human adopters [83].

**VI – Critical Quality Appraisal**

The twenty-sixth barrier we identified for development and implementation of AI tools and platforms to optimise the **critical quality appraisal** ES step regarded multi-stakeholder human validation [36] as crucial to avoid wrong system decisions [30], as for all previous ES steps. The twenty-seventh barrier regards Sarker and colleagues (2015) finding outlining that it was not possible to significantly improve the accuracy of AI tools and platforms with the then current available data. Nevertheless, at present, the LLMs landscape is rapidly changing with the recent release of several large open-sourced GPT models – such as Falcon, MosaicML, and LLaMA – and fine-tuning techniques – such as Low-Rank Adaptation (LoRA) and Quantized LoRA – with less computational and memory resources without compromising accuracy [83].

On the other hand, when it comes to semi-automatised **meta-analysis**, AI tools and platforms have accelerated certain ES steps, while still leaving more complex steps – i.e., **data extraction** and reviewer **selection** bias minimisation – to human input [36].

**VII – Summary/ Synthesis, Updating and Dissemination**

Regarding barriers to optimise ES, **summary/ synthesis** step, alongside **extraction** step as above mentioned, is the ES step where more LLMs have been developed and implemented. As such, the twenty-eighth development and implementation barrier we identified lies on the algorithm itself: since LLMs rely on the training data, the quality of the input data also plays a vital role in the effectiveness of LLMs (not only but specially) in qualitative research to avoid biases or inaccuracies (hallucinations) from noisy data [23, 27, 121]. In this sense, non-textual data, such as tables and figures in Cochrane Reviews, may increase the complexity and cause errors in the synthesis step [113] – the twenty-ninth barrier identified. Such errors can be categorised into: a) *misinterpretation* – i.e., overly convincing or uncertain summaries, potentially leading readers to rely too heavily on the accuracy of the presented information, further causing *contradiction* (discrepancy between the conclusions drawn from the medical evidence results and the summary) and *certainty illusion* (inconsistency in the degree of certainty between the summary and the source document); b) *fabricated* – i.e., when a statement appears in a summary, but no evidence from the source document can be found to support or refute the statement; c) *attribute* – i.e., any errors on non-key elements in the review question (i.e., PICO) which may arise under: c.1) *fabricated attribute* (not referenced in the source document); c.2) *omitted attribute* (leads to overgeneralization of conclusions); c.3) *distorted attribute* (incorrect data summary) [113]. All such errors may lead to LLMs generating factually inconsistent summaries, which lead to potentially harmful misinformation. Therefore, although ChatGPT presents the lowest proportion of all types of errors, once again, human input still remains essential to adequately evaluate both quality and factuality of ES generated by LLMs [113], further underlying the urgent need for comprehensive training based on expert annotations, formal evaluations, and rigorous regulatory precautions to ensure alignment with clinical performance standards and prevent harmful outcomes [27].

The thirtieth crucial barrier to the widely implemented ChatGPT is the lack of recency, as GPT-3.5 and GPT-4 were trained mostly using text generated up to September 2021 [33]. Given the dynamically evolving nature of research and innovation across fields, including medicine, events that breach the training dataset threshold date, will inevitably lead models to provide poor-quality responses to related queries. Once again, consultation with healthcare experts remains essential, especially for (continuous) **updating** processes (as in Living ES). As such, peer-reviewed scientific journals quickly stopped granting ChatGPT authorship in **publications**, further suggesting that the technology should, instead, be included as methodological tools assisting human-made work [33, 120]. On the other hand, since there is no current alternative to human creativity, scientific curiosity, and accountability, incorporating LLMs as valuable tools to optimise clinical research output while preserving and empowering human input in scientific publications is likely to revolutionise the biomedical industry, especially by means of increased efficiency and productivity [120]. As such, existing models built or fine-tuned with clinical text include ClinicalBERT, Med-PaLM 2 and GatorTron, which have collectively outperformed various general LLMs in biomedical NLP tasks [33].

### **References**

1. Tricco AC, Lillie E, Zarin W, et al (2018) PRISMA Extension for Scoping Reviews (PRISMA-ScR): Checklist and Explanation. Ann Intern Med 169:467–473

2. Levac D, Colquhoun H, O’Brien KK (2010) Scoping studies: advancing the methodology. Implement Sci. 5:

3. Peters MDJ, Godfrey CM, Khalil H, McInerney P, Parker D, Soares CB (2015) Guidance for conducting systematic scoping reviews. Int J Evid Based Healthc 13:141–146

4. Peters MDJ, Godfrey C, McInerney P, BaldiniSoares C, Khalil H, Parker D (2017) Chapter 11 - Scoping reviews. Adelaide, Australia

5. Jimenez RC, Lee T, Rosillo N, et al (2022) Machine learning computational tools to assist the performance of systematic reviews: A mapping review. BMC Med Res Methodol 22:322

6. Aljaber B, Martinez D, Stokes N, Bailey J (2011) Improving MeSH classification of biomedical articles using citation contexts. J Biomed Inform 44:881–896

7. Chai KEK, Lines RLJ, Gucciardi DF, Ng L (2021) Research Screener: a machine learning tool to semi-automate abstract screening for systematic reviews. Syst Rev. https://doi.org/10.1186/s13643-021-01635-3

8. Coiera E, Liu SD (2022) Evidence synthesis, digital scribes, and translational challenges for artificial intelligence in healthcare. Cell Rep Med. https://doi.org/10.1016/j.xcrm.2022.100860

9. Sarker A, Mollá D, Paris C (2015) Automatic evidence quality prediction to support evidence-based decision making. Artif Intell Med 64:89–103

10. Surian D, Dunn AG, Orenstein L, Bashir R, Coiera E, Bourgeois FT (2018) A shared latent space matrix factorisation method for recommending new trial evidence for systematic review updates. J Biomed Inform 79:32–40

11. Thomas J, McDonald S, Noel-Storr A, Shemilt I, Elliott J, Mavergames C, Marshall IJ (2021) Machine learning reduced workload with minimal risk of missing studies: development  and evaluation of a randomized controlled trial classifier for Cochrane Reviews. J Clin Epidemiol 133:140–151

12. Tsafnat G, Glasziou P, Karystianis G, Coiera E (2018) Automated screening of research studies for systematic reviews using study  characteristics. Syst Rev 7:64

13. Westgate MJ (2019) revtools: An R package to support article screening for evidence synthesis. Res Synth Methods 10:606–614

14. Khalil H, Ameen D, Zarnegar A (2022) Tools to support the automation of systematic reviews: a scoping review. J Clin Epidemiol 144:22–42

15. Tsafnat G, Glasziou P, Choong MK, Dunn A, Galgani F, Coiera E (2014) Systematic review automation technologies. Syst Rev. https://doi.org/10.1186/2046-4053-3-74

16. Beller E, Clark J, Tsafnat G, et al (2018) Making progress with the automation of systematic reviews: Principles of the International Collaboration for the Automation of Systematic Reviews (ICASR). Syst Rev. https://doi.org/10.1186/s13643-018-0740-7

17. Thomas J, Noel-Storr A, Marshall I, et al (2017) Living systematic reviews: 2. Combining human and machine effort. J Clin Epidemiol 91:31–37

18. Tsafnat G, Dunn A, Glasziou P, Coiera E (2012) The automation of systematic reviews. BMJ (Online). https://doi.org/10.1136/bmj.f139

19. Feng Y, Liang S, Zhang Y, Chen S, Wang Q, Huang T, Sun F, Liu X, Zhu H, Pan H (2022) Automated medical literature screening using artificial intelligence: A systematic review and meta-analysis. Journal of the American Medical Informatics Association 29:1425–1432

20. Wang JC, Su GD, Wan CR, Huang XW, Sun LL (2020) A Keyword-Based Literature Review Data Generating Algorithm-Analyzing a Field from Scientific Publications. SYMMETRY-BASEL. https://doi.org/10.3390/sym12060903 WE  - Science Citation Index Expanded (SCI-EXPANDED)

21. Yang X, Tang H, Dongye X, Chen G (2018) Exploration of meta analysis automation. In: 2018 International Conference on Network Infrastructure and Digital Content (IC-NIDC). IEEE, pp 218–222

22. Rajadhyax A, Moon D, Bhagat A, et al (2022) MSR100 Applicability of Artificial Intelligence in Targeted Literature Review. Value in Health 25:S369–S369

23. Baviskar D, Ahirrao S, Potdar V, Kotecha K (2021) Efficient Automated Processing of the Unstructured Documents Using Artificial Intelligence: A Systematic Literature Review and Future Directions. IEEE ACCESS 9:72894–72936

24. Matsui K, Utsumi T, Aoki Y, Maruki T, Takeshima M, Yoshikazu T (2023) Large Language Model Demonstrates Human-Comparable Sensitivity in Initial Screening of Systematic Reviews: A Semi-Automated Strategy Using GPT-3.5. Available at SSRN 4520426

25. Tsubota T, Bollegala D, Zhao Y, Jin Y, Kozu T (2022) Improvement of intervention information detection for automated clinical  literature screening during systematic review. J Biomed Inform 134:104185

26. Feng LY, Chiam YK, Lo SK (2017) Text-mining Techniques and Tools for Systematic Literature Reviews: A Systematic Literature Review. 2017 24TH ASIA-PACIFIC SOFTWARE ENGINEERING CONFERENCE (APSEC 2017) 41–50

27. Temsah O, Khan SA, Chaiah Y, et al (2023) Overview of Early ChatGPT’s Presence in Medical Literature: Insights From a  Hybrid Literature Review by ChatGPT and Human Experts. Cureus 15:e37281–e37281

28. Susnjak T (2023) PRISMA-DFLLM: An extension of PRISMA for systematic literature reviews using domain-specific finetuned large language models. arXiv preprint arXiv:2306.14905

29. Xiong Z, Liu T, Tse G, Gong M, Gladding PA, Smaill BH, Stiles MK, Gillis AM, Zhao J (2018) A machine learning aided systematic review and meta-analysis of the relative risk of atrial fibrillation in patients with diabetes mellitus. Front Physiol. https://doi.org/10.3389/fphys.2018.00835

30. Afzal M, Hussain M, Malik KM, Lee S (2019) Impact of Automatic Query Generation and Quality Recognition Using Deep Learning to Curate Evidence From Biomedical Literature: Empirical Study. JMIR Med Inform 7:e13430

31. Afzal M, Alam F, Malik KM, Malik GM (2020) Clinical Context–Aware Biomedical Text Summarization Using Deep Neural Network: Model Development and Validation. J Med Internet Res 22:e19810

32. Aum S, Choe S (2021) srBERT: automatic article classification model for systematic review using BERT. Syst Rev. https://doi.org/10.1186/s13643-021-01763-w

33. Thirunavukarasu AJ, Ting DSJ, Elangovan K, Gutierrez L, Tan TF, Ting DSW (2023) Large language models in medicine. Nat Med 29:1930–1940

34. Kaiser K, Miksch S (2009) Versioning computer-interpretable guidelines: semi-automatic modeling of “Living  Guidelines” using an information extraction method. Artif Intell Med 46:55–66

35. Kusa W, Hanbury A, Knoth P (2022) Automation of Citation Screening for Systematic Literature Reviews Using Neural Networks: A Replicability Study. ADVANCES IN INFORMATION RETRIEVAL, PT I 13185:584–598

36. Ajiji P, Cottin J, Picot C, Uzunali A, Ripoche E, Cucherat M, Maison P (2022) Feasibility study and evaluation of expert opinion on the semi-automated meta-analysis and the conventional meta-analysis. Eur J Clin Pharmacol 78:1177–1184

37. Martenot V, Masdeu V, Cupe J, Gehin F, Blanchon M, Dauriat J, Horst A, Renaudin M, Girard P, Zucker J-D (2022) LiSA: an assisted literature search pipeline for detecting serious adverse drug  events with deep learning. BMC Med Inform Decis Mak 22:338

38. Kempf S, Krug M, Puppe F (2023) KIETA: Key-insight extraction from scientific tables. APPLIED INTELLIGENCE 53:9513–9530

39. Nur S, Adams CE, Brailsford DF (2016) Using built-in functions of Adobe Acrobat Pro DC to help the selection process in systematic reviews of randomised trials. Systematic reviews 5 (1) (no pagination), 2016 Article number: 33 Date of publication: 18 feb 2016. https://doi.org/10.1186/s13643-016-0207-7

40. Barnickel T, Weston J, Collobert R, Mewes H-W, Stümpflen V (2009) Large scale application of neural network based semantic role labeling for automated relation extraction from biomedical texts. PLoS One. https://doi.org/10.1371/journal.pone.0006393

41. Perez-Iratxeta C, Bork P, Andrade MA (2001) XplorMed: A tool for exploring MEDLINE abstracts. Trends Biochem Sci 26:573–575

42. Goetz T, von der Lieth C-W (2005) PubFinder: A tool for improving retrieval rate of relevant PubMed abstracts. Nucleic Acids Res 33:W774–W778

43. Denzler T, Enders MR, Akello P, Syst AI (2021) Towards a Semi-Automated Approach for Systematic Literature Reviews Completed Research. DIGITAL INNOVATION AND ENTREPRENEURSHIP (AMCIS 2021)

44. Burgard T, Bittermann A (2023) Reducing Literature Screening Workload With Machine Learning A Systematic Review of Tools and Their Performance. ZEITSCHRIFT FUR PSYCHOLOGIE-JOURNAL OF PSYCHOLOGY 231:3–15

45. Li J, Dada A, Kleesiek J, Egger J (2023) ChatGPT in Healthcare: A Taxonomy and Systematic Review. medRxiv 2023

46. Adam GP, Pappas D, Papageorgiou H, Evangelou E, Trikalinos TA (2022) A novel tool that allows interactive screening of PubMed citations showed promise for the semi-automation of identification of Biomedical Literature. J Clin Epidemiol 150:63–71

47. Fleuren WWM, Alkema W (2015) Application of text mining in the biomedical domain. Methods 74:97–106

48. van Altena AJ, Spijker R, Leeflang MMG, Olabarriaga SD (2021) Training sample selection: Impact on screening automation in diagnostic test accuracy reviews. Res Synth Methods 12:831–841

49. Van De Schoot R, De Bruin J, Schram R, Zahedi P, De Boer J, Weijdema F, Kramer B, Huijts M, Hoogerwerf M, Ferdinands G (2021) An open source machine learning framework for efficient and transparent systematic reviews. Nat Mach Intell 3:125–133

50. Serban R, ten Teije A, van Harmelen F, Marcos M, Polo-Conde C (2007) Extraction and use of linguistic patterns for modelling medical guidelines. Artif Intell Med 39:137–149

51. Soboczenski F, Trikalinos TA, Kuiper J, Bias RG, Wallace BC, Marshall IJ (2019) Machine learning to help researchers evaluate biases in clinical trials: a  prospective, randomized user study. BMC Med Inform Decis Mak 19:96

52. Jardim PSJ, Rose CJ, Ames HM, Echavez JFM, de Velde S, Muller AE (2022) Automating risk of bias assessment in systematic reviews: a real-time mixed  methods comparison of human researchers to a machine learning system. BMC Med Res Methodol 22:167

53. Røst TB, Slaughter L, Nytro O, Muller AE, Vist GE, Røst TB, Slaughter L, Nytrø Ø, Muller AE, Vist GE (2021) Using neural networks to support high-quality evidence mapping. BMC Bioinformatics 22:496

54. de la Torre-lopez J, Ramirez A, Romero JR (2023) Artificial intelligence to automate the systematic review of scientific literature. COMPUTING. https://doi.org/10.1007/s00607-023-01181-x

55. Pérez-Pérez M, Ferreira T, Lourenço A, Igrejas G, Fdez-Riverola F (2022) Boosting biomedical document classification through the use of domain entity recognizers and semantic ontologies for document representation: The case of gluten bibliome. Neurocomputing 484:223–237

56. Tercero-Hidalgo JR, Khan KS, Bueno-Cavanillas A, Fernández-López R, Huete JF, Amezcua-Prieto C, Zamora J, Fernández-Luna JM (2022) Artificial intelligence in COVID-19 evidence syntheses was underutilized, but impactful: a methodological study. J Clin Epidemiol 148:124–134

57. Müller H, Pachnanda S, Pahl F, Rosenqvist C, IEEE (2022) The application of artificial intelligence on different types of literature reviews - A comparative study. 2022 INTERNATIONAL CONFERENCE ON APPLIED ARTIFICIAL INTELLIGENCE (ICAPAI) 38–44

58. Park J, Djelassi M, Chima D, Hernandez R, Poroshin V, Iliescu A-M, Domalik D, Southall N (2023) Validation of a Natural Language Machine Learning Model for Safety Literature Surveillance. Drug Saf. https://doi.org/10.1007/s40264-023-01367-4

59. Dhrangadhariya A, Hilfiker R, Schaer R, Müller H (2020) Machine Learning Assisted Citation Screening for Systematic Reviews. Stud Health Technol Inform 270:302–306

60. Orel E, Ciglenecki I, Thiabaud A, Temerev A, Calmy A, Keiser O, Merzouki A (2023) An Automated Literature Review Tool (LiteRev) for Streamlining and Accelerating Research Using Natural Language Processing and Machine Learning: Descriptive Performance Evaluation Study. J Med Internet Res. https://doi.org/10.2196/39736

61. Queiros L, Witzmann A, Sumner M, Wehler P, Baehrens D, Abogunrin S (2022) POSB317 Machines As a Second Reviewer in Systematic Literature Reviews. Value in Health 25:S205–S206

62. Queiros L, Mearns ES, Ademisoye E, McCarvil M, Alarcão J, Garcia MJ, Abogunrin S (2022) MSR22 Is Artificial Intelligence Replacing Humans in Systematic Literature Reviews? a Systematic Literature Review. Value in Health 25:S522–S522

63. Robson B (2016) Studies in using a universal exchange and inference language for evidence based medicine. Semi-automated learning and reasoning for PICO methodology, systematic review, and environmental epidemiology. Comput Biol Med 79:299–323

64. Olorisade BK, Brereton P, Andras P (2019) The use of bibliography enriched features for automatic citation screening. J Biomed Inform. https://doi.org/10.1016/j.jbi.2019.103202

65. Ananiadou S, Rea B, Okazaki N, Procter R, Thomas J (2009) Supporting systematic reviews using text mining. Soc Sci Comput Rev 27:509–523

66. Aliyu MB, Iqbal R, James A (2018) The Canonical Model of Structure for Data Extraction in Systematic Reviews of Scientific Research Articles. In: 2018 Fifth International Conference on Social Networks Analysis, Management and Security (SNAMS). IEEE, pp 264–271

67. Stansfield C, Thomas J, Kavanagh J (2013) “Clustering” documents automatically to support scoping reviews of research: a  case study. Res Synth Methods 4:230–241

68. Kontonatsios G, Brockmeier AJ, Przybyła P, McNaught J, Mu T, Goulermas JY, Ananiadou S (2017) A semi-supervised approach using label propagation to support citation screening. J Biomed Inform 72:67–76

69. Mo Y, Kontonatsios G, Ananiadou S (2015) Supporting systematic reviews using LDA-based document representations. Syst Rev 4:1–12

70. Bannach-Brown A, Przybyła P, Thomas J, Rice ASC, Ananiadou S, Liao J, Macleod MR (2019) Machine learning algorithms for systematic review: Reducing workload in a preclinical review of animal studies and reducing human screening error. Syst Rev. https://doi.org/10.1186/s13643-019-0942-7

71. Brassey J, Price C, Edwards J, Zlabinger M, Bampoulidis A, Hanbury A (2021) Developing a fully automated evidence synthesis tool for identifying, assessing and collating the evidence. BMJ Evid Based Med 26:24–27

72. Schmidt L, Finnerty Mutlu AN, Elmore R, Olorisade BK, Thomas J, Higgins JPT (2023) Data extraction methods for systematic review (semi)automation: Update of a  living systematic review. F1000Res 10:401

73. Robinson A, Thorne W, Wu BP, Pandor A, Essat M, Stevenson M, Song X (2023) Bio-SIEVE: Exploring Instruction Tuning Large Language Models for Systematic Review Automation. arXiv preprint arXiv:2308.06610

74. Sutton A, O’Keefe H, Johnson EE, Marshall C (2023) A mapping exercise using automated techniques to develop a search strategy to  identify systematic review tools. Res Synth Methods 14:874–881

75. Marshall IJ, Wallace BC (2019) Toward systematic review automation: A practical guide to using machine learning tools in research synthesis. Syst Rev. https://doi.org/10.1186/s13643-019-1074-9

76. Harrison H, Griffin SJ, Kuhn I, Usher-Smith JA (2020) Software tools to support title and abstract screening for systematic reviews in healthcare: an evaluation. BMC Med Res Methodol 20:1–12

77. Park SE, Thomas J (2018) Evidence synthesis software. Evid Based Med 23:140–141

78. Hair K, Wilson E, Wong C, Tsang A, Macleod M, Bannach-Brown A (2023) Systematic online living evidence summaries: emerging tools to accelerate evidence synthesis. Clin Sci 137:773–784

79. Halfpenny N, Alleman C, Eaton J, van Vliet M (2019) PNS335 USING MACHINE LEARNING FOR EFFICIENCY IMPROVEMENTS IN SYSTEMATIC LITERATURE REVIEWS OF CLINICAL EFFICACY AND SAFETY. Value in Health 22:S821–S821

80. Li Z, Belkadi S, Micheletti N, Han L, Shardlow M, Nenadic G (2023) Large Language Models and Control Mechanisms Improve Text Readability of Biomedical Abstracts. arXiv preprint arXiv:2309.13202

81. Nashwan AJ, Jaradat JH (2023) Streamlining Systematic Reviews: Harnessing Large Language Models for Quality Assessment and Risk-of-Bias Evaluation. Cureus 15:

82. Ouzzani M, Hammady H, Fedorowicz Z, Elmagarmid A (2016) Rayyan—a web and mobile app for systematic reviews. Syst Rev 5:1–10

83. Tian S, Jin Q, Yeganova L, Lai P-T, Zhu Q, Chen X, Yang Y, Chen Q, Kim W, Comeau DC (2023) Opportunities and Challenges for ChatGPT and Large Language Models in Biomedicine and Health. arXiv preprint arXiv:2306.10070

84. Raza Abidi SS, Kershaw M, Milios E (2005) Augmenting GEM-encoded clinical practice guidelines with relevant best evidence autonomously retrieved from MEDLINE. Health Informatics J 11:95–110

85. Kiritchenko S, De Bruijn B, Carini S, Martin J, Sim I (2010) ExaCT: automatic extraction of clinical trial characteristics from journal publications. BMC Med Inform Decis Mak 10:1–17

86. Matwin S, Kouznetsov A, Inkpen D, Frunza O, O’Blenis P (2010) A new algorithm for reducing the workload of experts in performing systematic reviews. Journal of the American Medical Informatics Association 17:446–453

87. Tetzlaff J, Murad MH, Wang Z (2020) AI4 CAN WE DECREASE THE SCREENING BURDEN IN SYSTEMATIC REVIEWS? PERFORMANCE OF TWO NATURAL LANGUAGE PROCESSORS TO EXCLUDE RECORDS. Value in Health 23:S1–S2

88. Tetzlaff J, Cadarette SM, O’Blenis P, Ruiz K (2019) PNS15 PRAGMATIC ARTIFICIAL INTELLIGENCE-BASED REFERENCE SCREENING IN SYSTEMATIC REVEIWS. ARE TWO ROBOTS BETTER THAN ONE? Value in Health 22:S290–S290

89. Kwabena AE, Wiafe O-B, John B-D, Bernard A, Boateng FAF (2023) An automated method for developing search strategies for systematic review using Natural Language Processing (NLP). MethodsX. https://doi.org/10.1016/j.mex.2022.101935

90. Guo E, Gupta M, Deng J, Park Y-J, Paget M, Naugler C (2023) Automated Paper Screening for Clinical Reviews Using Large Language Models. arXiv preprint arXiv:2305.00844

91. Gates A, Johnson C, Hartling L (2018) Technology-assisted title and abstract screening for systematic reviews: a  retrospective evaluation of the Abstrackr machine learning tool. Syst Rev 7:45

92. Gates A, Guitard S, Pillay J, Elliott SA, Dyson MP, Newton AS, Hartling L (2019) Performance and usability of machine learning for screening in systematic reviews: A comparative evaluation of three tools. Syst Rev. https://doi.org/10.1186/s13643-019-1222-2

93. Gates A, Gates M, Sebastianski M, Guitard S, Elliott SA, Hartling L (2020) The semi-automation of title and abstract screening: a retrospective exploration  of ways to leverage Abstrackr’s relevance predictions in systematic and rapid reviews. BMC Med Res Methodol 20:139

94. Hamel C, Kelly SE, Thavorn K, Rice DB, Wells GA, Hutton B (2020) An evaluation of DistillerSR’s machine learning-based prioritization tool for  title/abstract screening - impact on reviewer-relevant outcomes. BMC Med Res Methodol 20:256

95. Gates A, Vandermeer B, Hartling L (2018) Technology-assisted risk of bias assessment in systematic reviews: a prospective cross-sectional evaluation of the RobotReviewer machine learning tool. J Clin Epidemiol 96:54–62

96. Wagner G, Lukyanenko R, Paré G (2022) Artificial intelligence and the conduct of literature reviews. JOURNAL OF INFORMATION TECHNOLOGY 37:209–226

97. Kamdar BB, Shah PA, Sakamuri S, Kamdar BS, Oh J (2015) A NOVEL SEARCH BUILDER to EXPEDITE SEARCH STRATEGIES for SYSTEMATIC REVIEWS. Int J Technol Assess Health Care 31:51–53

98. Wallace BC, Small K, Brodley CE, Lau J, Schmid CH, Bertram L, Lill CM, Cohen JT, Trikalinos TA (2012) Toward modernizing the systematic review pipeline in genetics: Efficient updating via data mining. Genetics in Medicine 14:663–669

99. Vasuki V, Cohen T (2010) Reflective random indexing for semi-automatic indexing of the biomedical literature. J Biomed Inform 43:694–700

100. Grames EM, Stillman AN, Tingley MW, Elphick CS (2019) An automated approach to identifying search terms for systematic reviews using keyword co-occurrence networks. Methods Ecol Evol 10:1645–1654

101. Allot A, Lee K, Chen Q, Luo L, Lu Z (2021) LitSuggest: A web-based system for literature recommendation and curation using machine learning. Nucleic Acids Res 49:W352–W358

102. Timsina P, El-Gayar O, Liu J, Syst AI (2015) Active Learning for the Automation of Medical Systematic Review Creation. AMCIS 2015 PROCEEDINGS

103. Wallace BC, Small K, Brodley CE, Lau J, Trikalinos TA (2012) Deploying an interactive machine learning system in an evidence-based practice center: abstrackr. Proceedings of the 2nd ACM SIGHIT international health informatics symposium 819–824

104. Reddy SM, Patel S, Weyrich M, Fenton J, Viswanathan M (2020) Comparison of a traditional systematic review approach with review-of-reviews and semi-automation as strategies to update the evidence. Syst Rev. https://doi.org/10.1186/s13643-020-01450-2

105. Bekhuis T, Tseytlin E, Mitchell KJ, Demner-Fushman D (2014) Feature engineering and a proposed decision-support system for systematic reviewers of medical evidence. PLoS One. https://doi.org/10.1371/journal.pone.0086277

106. Gartlehner G, Wagner G, Lux L, Affengruber L, Dobrescu A, Kaminski-Hartenthaler A, Viswanathan M (2019) Assessing the accuracy of machine-assisted abstract screening with DistillerAI: A user study. Syst Rev. https://doi.org/10.1186/s13643-019-1221-3

107. Schneider J, Hoang L, Kansara Y, Cohen AM, Smalheiser NR (2022) Evaluation of publication type tagging as a strategy to screen randomized controlled trial articles in preparing systematic reviews. JAMIA Open. https://doi.org/10.1093/jamiaopen/ooac015

108. Cohen AM, Hersh WR, Peterson K, Yen P-Y (2006) Reducing workload in systematic review preparation using automated citation classification. Journal of the American Medical Informatics Association 13:206–219

109. Cohen AM, Smalheiser NR, McDonagh MS, Yu C, Adams CE, Davis JM, Yu PS (2015) Automated confidence ranked classification of randomized controlled trial articles: An aid to evidence-based medicine. Journal of the American Medical Informatics Association 22:707–717

110. Walker VR, Schmitt CP, Wolfe MS, et al (2022) Evaluation of a semi-automated data extraction tool for public health literature-based reviews: Dextr. Environ Int. https://doi.org/10.1016/j.envint.2021.107025

111. Evans DA, Hersh WR, Monarch IA, Lefferts RG, Handerson SK (1991) Automatic indexing of abstracts via natural-language processing using a simple thesaurus. Medical Decision Making 11:S108–S115

112. Yu B (2022) Evaluating pre-trained language models on multi-document summarization for literature reviews. Proceedings of the Third Workshop on Scholarly Document Processing 188–192

113. Tang L, Sun Z, Idnay B, Nestor JG, Soroush A, Elias PA, Xu Z, Ding Y, Durrett G, Rousseau JF (2023) Evaluating large language models on medical evidence summarization. NPJ Digit Med 6:158

114. Bui DDA, Del Fiol G, Jonnalagadda S (2016) PDF text classification to leverage information extraction from publication reports. J Biomed Inform 61:141–148

115. Elamin MB, Flynn DN, Bassler D, Briel M, Alonso-Coello P, Karanicolas PJ, Guyatt GH, Malaga G, Furukawa TA, Kunz R (2009) Choice of data extraction tools for systematic reviews depends on resources and review complexity. J Clin Epidemiol 62:506–510

116. States DJ, Ade AS, Wright ZC, Bookvich A V, Athey BD (2009) MiSearch adaptive pubMed search tool. Bioinformatics 25:974–976

117. Wallace BC, Dahabreh IJ, Schmid CH, Lau J, Trikalinos TA (2013) Modernizing the systematic review process to inform comparative effectiveness: Tools and methods. J Comp Eff Res 2:273–282

118. Cohen AM, Ambert K, McDonagh M (2009) Cross-Topic Learning for Work Prioritization in Systematic Review Creation and Update. Journal of the American Medical Informatics Association 16:690–704

119. Cowie K, Rahmatullah A, Hardy N, Holub K, Kallmes K (2022) Web-Based Software Tools for Systematic Literature Review in Medicine: Systematic  Search and Feature Analysis. JMIR Med Inform 10:e33219–e33219

120. Diaz Milian R, Moreno Franco P, Freeman WD, Halamka JD (2023) Revolution or Peril? The Controversial Role of Large Language Models in Medical Manuscript Writing. Mayo Clin Proc 98:1444–1448

121. Tai RH, Bentley LR, Xia X, Sitt JM, Fankhauser SC, Chicas-Mosier AM, Monteith BG (2023) Use of large language models to aid analysis of textual data. bioRxiv 2023–2027

122. Van Veen D, Van Uden C, Blankemeier L, Delbrouck J-B, Aali A, Bluethgen C, Pareek A, Polacin M, Collins W, Ahuja N (2023) Clinical text summarization: adapting large language models can outperform human experts. arXiv preprint arXiv:2309.07430

123. Felizardo KR, Salleh N, Martins RM, Mendes E, MacDonell SG, Maldonado JC (2011) Using visual text mining to support the study selection activity in systematic literature reviews. 2011 international symposium on empirical software engineering and measurement 77–86

124. Tsunoda DF, Moreira PSD, Guimaraes AJR (2020) Machine learning and automated systematic literature review: a systematic review. REVISTA TECNOLOGIA E SOCIEDADE 16:337–354

125. dos Santos AO, da Silva ES, Couto LM, Reis GVL, Belo VS, Santos ÁOD, da Silva ES, Couto LM, Reis GVL, Belo VS (2023) The use of artificial intelligence for automating or semi-automating biomedical literature analyses: A scoping review. J Biomed Inform. https://doi.org/10.1016/j.jbi.2023.104389

126. Giglio A Del, Costa MUP da (2023) The use of artificial intelligence to improve the scientific writing of non-native english speakers. Rev Assoc Med Bras (1992, Impr) 69:e20230560–e20230560

127. Roco-Videla Á, Aguilera-Eguía R, Olguín-Barraza M, Flores-Fernández C (2023) El papel de la inteligencia artificial en las revisiones sistemáticas: implicaciones y desafíos para la divulgación científica TT  - The role of artificial intelligence in systematic reviews: implications and challenges for scientific dissemination. Angiol (Barcelona) 75:344–345

128. Schmidt L, Sinyor M, Webb RT, Marshall C, Knipe D, Eyles EC, John A, Gunnell D, Higgins JPT (2023) A narrative review of recent tools and innovations toward automating living  systematic reviews and evidence syntheses. Z Evid Fortbild Qual Gesundhwes 181:65–75

129. Schmidt L, Olorisade BK, McGuinness LA, Thomas J, Higgins JPT (2020) Data extraction methods for systematic review (semi)automation: A living review protocol. F1000Res. https://doi.org/10.12688/f1000research.22781.2

130. Howard BE, Phillips J, Miller K, Tandon A, Mav D, Shah MR, Holmgren S, Pelch KE, Walker V, Rooney AA (2016) SWIFT-Review: a text-mining workbench for systematic review. Syst Rev 5:1–16

131. Alshami A, Elsayed M, Ali E, Eltoukhy AEE, Zayed T (2023) Harnessing the Power of ChatGPT for Automating Systematic Review Process: Methodology, Case Study, Limitations, and Future Directions. SYSTEMS. https://doi.org/10.3390/systems11070351 WE  - Social Science Citation Index (SSCI)

132. O’Keefe H, Rankin J, Wallace SA, Beyer F (2023) Investigation of text-mining methodologies to aid the construction of search  strategies in systematic reviews of diagnostic test accuracy-a case study. Res Synth Methods 14:79–98

133. Marshall IJ, Johnson BT, Wang Z, Rajasekaran S, Wallace BC (2020) Semi-Automated evidence synthesis in health psychology: current methods and  future prospects. Health Psychol Rev 14:145–158

134. Pham B, Jovanovic J, Bagheri E, et al (2021) Text mining to support abstract screening for knowledge syntheses: a semi-automated workflow. Syst Rev. https://doi.org/10.1186/s13643-021-01700-x

135. Rathbone J, Hoffmann T, Glasziou P (2015) Faster title and abstract screening? Evaluating Abstrackr, a semi-automated online screening program for systematic reviewers. Syst Rev 4:1–7

136. PAHO (2024) Q&A on artificial intelligence for supporting public health: Reference tool to support the exchange of information and promote open conversations and debates. Washington

137. WHO (2021) Ethics and governance of artificial intelligence for health: WHO guidance. Geneva

138. WHO (2023) WHO calls for safe and ethical AI for health. Geneva

139. Raiaan MAK, Mukta MSH, Fatema K, Fahad NM, Sakib S, Mim M, Jannat M, Ahmad J, Ali ME, Azam S (2023) A Review on Large Language Models: Architectures, Applications, Taxonomies, Open Issues and Challenges.

140. Minssen T, Vayena E, Cohen IG (2023) The Challenges for Regulating Medical Use of ChatGPT and Other Large Language Models. JAMA 330:315

141. Prainsack B, Forgó N (2024) New AI regulation in the EU seeks to reduce risk without assessing public benefit. Nat Med 30:1235–1237

142. Andreoletti M, Haller L, Vayena E, Blasimme A (2024) Mapping the ethical landscape of digital biomarkers: A scoping review. PLOS Digital Health 3:e0000519

143. Blasimme A, Vayena E (2019) The Ethics of AI in Biomedical Research, Patient Care and Public Health. SSRN Electronic Journal. https://doi.org/10.2139/ssrn.3368756

144. Panch T, Pearson-Stuttard J, Greaves F, Atun R (2019) Artificial intelligence: opportunities and risks for public health. Lancet Digit Health 1:e13–e14

### **GANTT Chart for Scoping Review Development**

| **Scoping Review Stage** | **Oct/23** | **Nov/23** | **Dec/23** | **Jan/24** | **Feb/24** | **Mar/24** | **Apr/24** | **May/24** | **Jun/24** | **Jul-Oct/24** |
| --- | --- | --- | --- | --- | --- | --- | --- | --- | --- | --- |
| Protocol Development |  |  |  |  |  |  |  |  |  |  |
| Conduct initial searches to refine protocol |  |  |  |  |  |  |  |  |  |  |
| Conduct searches and eliminate duplications |  |  |  |  |  |  |  |  |  |  |
| Screening by Reviewer Duos 1 and 2 |  |  |  |  |  |  |  |  |  |  |
| Data extraction by Reviewer Duos 1 and 2 |  |  |  |  |  |  |  |  |  |  |
| Findings assessment and synthesis |  |  |  |  |  |  |  |  |  |  |
| Writing up paper (and search update) |  |  |  |  |  |  |  |  |  |  |
| Submit paper for publication |  |  |  |  |  |  |  |  |  |  |
